# The Pathways between Cortisol-Related Regulation Genes and PTSD Psychotherapy

**DOI:** 10.3390/healthcare8040376

**Published:** 2020-10-01

**Authors:** Ivone Castro-Vale, Davide Carvalho

**Affiliations:** 1 Medical Psychology Unit, Department of Clinical Neurosciences and Mental Health, Faculty of Medicine, University of Porto, Al. Prof. Hernâni Monteiro, 4200-319 Porto, Portugal; 2 i3S-Institute for Research and Innovation in Health, University of Porto, Rua Alfredo Allen, 208, 4200-135 Porto, Portugal; 3 Department of Endocrinology, Diabetes and Metabolism, São João Hospital University Centre, Faculty of Medicine, University of Porto, Al. Prof. Hernâni Monteiro, 4200-319 Porto, Portugal; davideccarvalho@gmail.com

**Keywords:** posttraumatic stress disorder, psychotherapy, glucocorticoids, cortisol, glucocorticoid receptor, *NR3C1*, *FKBP5*

## Abstract

Post-traumatic stress disorder (PTSD) only develops after exposure to a traumatic event in some individuals. PTSD can be chronic and debilitating, and is associated with co-morbidities such as depression, substance use, and cardiometabolic disorders. One of the most important pathophysiological mechanisms underlying the development of PTSD and its subsequent maintenance is a dysfunctional hypothalamic–pituitary–adrenal (HPA) axis. The corticotrophin-releasing hormone, cortisol, glucocorticoid receptor (GR), and their respective genes are some of the mediators of PTSD’s pathophysiology. Several treatments are available, including medication and psychotherapies, although their success rate is limited. Some pharmacological therapies based on the HPA axis are currently being tested in clinical trials and changes in HPA axis biomarkers have been found to occur in response not only to pharmacological treatments, but also to psychotherapy—including the epigenetic modification of the GR gene. Psychotherapies are considered to be the first line treatments for PTSD in some guidelines, even though they are effective for some, but not for all patients with PTSD. This review aims to address how knowledge of the HPA axis-related genetic makeup can inform and predict the outcomes of psychotherapeutic treatments.

## 1. Introduction

Post-traumatic stress disorder (PTSD) is a trauma- and stressor-related disorder which can only develop after the experience of a major traumatic event (TE) [1]. In the fifth Edition of the Diagnostic and Statistical Manual of Mental Disorders (DSM-5) [1], PTSD criteria were redefined and are now characterised by four symptom clusters, namely: re-experience of the TE; avoidance of stimuli associated with the TE; negative cognitions and mood; and alterations in arousal, all of which must last for more than one month and cause significant clinical distress or impairment in important areas of functioning. Although the experience of TEs is very frequent in the population, with a lifetime prevalence ranging from 64–90% [2,3], only around 10% of the population will develop the disorder [4]. PTSD development is highly dependent on the type of TE, the individual genetic makeup, and other risk factors, as is also the manifestation of different symptoms and the severity of PTSD [5,6].

When an individual experiences a TE, the stress-reactive systems are activated, which include the sympathoadrenomedullary drive and the hypothalamic–pituitary–adrenal (HPA) axis [7]. Individuals with a history of previous increased vulnerability to PTSD development (e.g., childhood adversities which modify the stress-reactive systems [8,9]) can experience PTSD symptoms immediately after the TE experience, or later on, while those with no such vulnerability can develop PTSD due to specific direct TE-related effects on the stress systems (e.g., epigenetic modifications or trauma interaction with specific single nucleotide polymorphisms (SNPs)).

The reason why only a limited number of people who experienced TEs develop PTSD has been subject to debate in the scientific community [10]. Gene/environment (G × E) interactions have been indicated as reliable mechanisms [11]. Indeed, it appears that vulnerability to develop PTSD can result from exposure to environmental adversity in cases of specific genetic makeup, such as, for example, SNPs and the epigenome [12]. TEs can also influence genetic expression through epigenetic mechanisms. Furthermore, exposure to other previous adverse experiences increases the risk of PTSD development [13], through epigenetic mechanisms [8]. SNPs can also interact with environmental exposure and epigenetic modifications to increase the risk of PTSD development [14].

A recent systematic review found that the examination of all the included PTSD studies consistently implicated DNA methylation and gene expression changes in the HPA axis and inflammatory genes in PTSD pathophysiology [15]. Indeed, genetic factors have been estimated to account for 30–46% of the variance in PTSD [16,17]. Being a stress-related disorder, HPA axis regulator genes have been extensively studied—such as the glucocorticoid receptor (GR) and FK506 binding protein 51 (FKBP5) genes [10]. Although the risk of developing PTSD is polygenetic, a recent meta-analysis found SNPs in these two genes to be associated with PTSD [18]. Furthermore, the GR gene (*NR3C1*) has been considered to be a candidate gene which underlies the neurobiological-related glucocorticoid (GC) processes involved in the development or maintenance of PTSD [12].

PTSD is a heterogeneous disorder and the determinants of different conditional risks for PTSD development can be categorised in several ways [19], including the type and burden of the TE and the sex and age of the exposed subject, which also determine the symptomatic expression, clinical trajectories, co-morbidity, and treatment response. Furthermore, the HPA axis regulation of the response to TE and stress is highly complex and is subject to a large degree of variability.

As a result, different clinical trajectories of PTSD development have been described in different trauma-exposed populations [10,20,21,22,23]. The longer-lasting prospective study found five classes of trajectories based on PTSD symptom severity, which were assessed using the Clinician-Administered PTSD Scale score, during the six years after traumatic injury. These are: chronic (4%), recovery (6%), worsening/recovery (8%), worsening (10%), and resilient (73%) [21]. In addition, PTSD can be very debilitating and is also associated with mental and somatic comorbidity [24,25], such as depression, substance use disorders, cardiovascular conditions, and endocrine and metabolic disorders, as well as with intergenerational transmission of depression, emotion dysregulation, and HPA axis dysfunction [26,27,28]. 

Several treatments have been indicated for patients with PTSD, which mainly include pharmacotherapy and psychotherapy. As pharmacotherapy approaches have considerable high rates of non-response, significant investment has been carried out in applying other therapies—particularly psychotherapies. Various psychotherapies have been indicated for the treatment of PTSD, with reported success being superior to that of the prescription of medication in several studies [29], although their effectiveness has been questioned [30]. Accordingly, there is a need to improve existing PTSD psychotherapeutic treatments, as almost two-thirds of patients receiving prolonged exposure therapy (PE) or cognitive processing therapy (CPT)—which are the two most widely-recommended first-line therapies—maintained the same diagnosis posttreatment [31].

It is becoming clearer that psychotherapeutic interventions must be individually tailored. Psychotherapeutic intervention in patients with PTSD has been associated with change in plasma cortisol [32], and more recently with changes in the epigenome [33,34]. Furthermore, SNPs in the GR and *FKBP5* genes and *FKBP5* epigenomic alterations predict psychotherapeutic outcomes [33,35,36]. Another ten genes, including a gene related with stress vulnerability (*ZFP57*), have also been recently associated with psychotherapeutic outcomes in a longitudinal study of genome-wide DNA methylation levels [34]. Successful treatment of PTSD patients with psychotherapy was accompanied by changes in DNA methylation.

In this paper we aim to review the extant knowledge regarding the interplay between the HPA axis regulatory genes and psychotherapeutic interventions for patients with PTSD, by taking into account the previously-reported HPA axis-related G × E interactions involved in PTSD pathophysiology, as well as the interactions between the genetic makeup and psychotherapy and the urgent need to improve patients’ psychotherapeutic outcomes. 

## 2. The HPA Axis

### 2.1. Neuroendocrine Regulation

The HPA axis is necessary for adaptation to stress. Physiological stimuli and stress activate the HPA axis (a three-organ hormonal cascade and feedback cycle) which leads to GC (cortisol in humans) secretion and release from the adrenal cortex (Box 1). Indeed, cortisol is both the primary GC which signals the stress response and the primary inhibitor of continuing HPA axis activity and of the autonomic stress responses [7].

Box 1 **The hypothalamic–pituitary–adrenal (HPA) axis.**

Both physiological stimuli and stress trigger the HPA axis leading to glucocorticoids (GCs, cortisol in humans) release from the adrenal cortex:

- Physiological homeostatic challenges involve, through sensory relays, the direct noradrenergic and peptidergic (originating mainly from the nucleus of the solitary tract) stimulation of the corticotrophin-releasing hormone (CRH) neurons which are located in the dorsomedial parvocellular (mp) division of the hypothalamic paraventricular nucleus (PVN) [37]. Further processing of the information includes glutamatergic, serotoninergic, and cytokine stimulation of the PVN. Under specific conditions, GABA can also stimulate the HPA axis at the PVN.
- Anticipatory responses involve the trans-synaptic activation of the HPA axis through the integration of multimodal sensory information, and the respective higher processing, which involves the ventral hippocampus, the medial prefrontal cortex, and the amygdala, among other limbic structures [7].
- Hypothalamic PVN is subject to regulation by several structures by way of glutamatergic and GABAergic neurons and the endocannabinoid system.
- The amygdala plays an important role in the defensive responses to learned threats. [38]. The lateral nucleus is important for the acquisition of aversive learning [39,40]. The aversive stimulus is recognised and processed in the amygdala’s basolateral complex, which connects with the central nucleus to stimulate the brainstem through CRH projections to the locus coeruleus [41] and the hypothalamus preparing the person to cope with the stimulus.
- GCs act through rapid non-genomic mechanisms in the basolateral amygdala through a membrane GC receptor (mGR), inducing the release of endocannabinoids from postsynaptic membranes, generating a retrograde feedback suppression on GABAergic neurons, and thereby driving the release of norepinephrine to the amygdala, which is required for emotional memory acquisition [42,43]. The prefrontal cortex and the hippocampus project to both the PVN and the amygdala in order to regulate the response to the aversive stimulus [7,44].
- CRH binds to the CRH type 1 receptor in the anterior pituitary, stimulating the release of the adrenocorticotrophic hormone (ACTH) into the bloodstream. Arginine vasopressin is co-released and it complements CRH actions in ACTH release.
- ACTH action on the adrenal cortex leads to cortisol production and release, which is potentiated by the sympathetic nervous system and cytokines.
- The GC response depends on the timing of the stressful event: if this event occurs during the ascending phase of the ultradian pulse, it tends to have a greater level of response than if it were to occur during the falling phase [7,45].
- Cortisol serves several functions, including mobilisation of energy to cope with the aversive stimuli, memory processing, immune system modulation, the termination of the response by active feedback processes, repressing all unnecessary activity—such as growth (emergency mode: when a person is faced with a traumatic event (TE) GCs inactivate all non-essential activities for survival [46]). Another important effect of GCs is to enhance the consolidation of the memories of fear and other strong emotions to facilitate coping with re-exposure [47].

Cortisol exerts crucial feedback effects on binding to GRs in the hypothalamus and the pituitary, which reduces the release of both corticotrophin-releasing hormone (CRH) and adrenocorticotrophic hormone (ACTH) [7]. GC-bound mGR releases endocannabinoids, which, in turn, inhibit the release of glutamate, which reduces the stimulation of the hypothalamic paraventricular nucleus (PVN) CRH neurons. GABAergic and neuropeptidergic inputs also play important roles in inhibiting CRH release. Transsynaptic GR mediated HPA axis inhibition also occurs in the hippocampus, as well as the prefrontal cortex and the hindbrain. These feedback effects are very important to stop the exposure to the catabolic actions of GC and the sympathetic nervous system effects. Norepinephrine is an important mediator of the central nervous system (CNS) and autonomic stress responses. Norepinephrine and CRH interact in the hypothalamus, locus coeruleus, and amygdala circuit, integrating autonomic and HPA axis responses to stress. 

**Figure 1:**
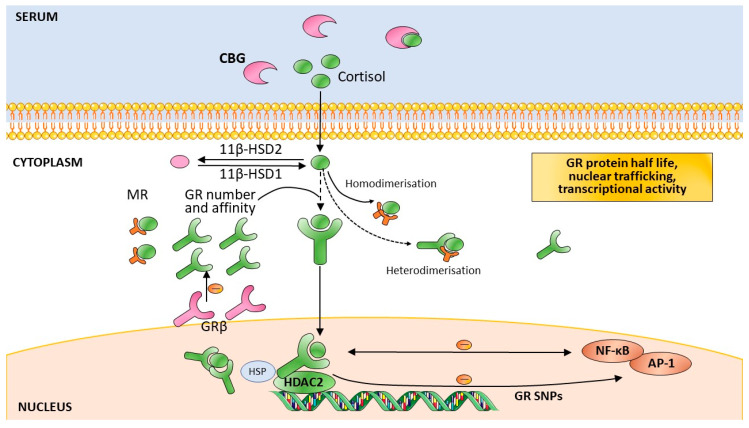
Modulators of the cellular action of glucocorticoids. These modulators include glucocorticoid availability, glucocorticoid receptor number and affinity, GRβ isoform expression, glucocorticoid receptor signalling, mineralocorticoid receptor signalling, glucocorticoid receptor–gene interaction, and glucocorticoid receptor single nucleotide polymorphisms. Abbreviations: 11β-HSD: 11-β-hydroxysteroid dehydrogenase; CBG: Corticosteroid-binding globulin; GR: glucocorticoid receptor; HSP: heat shock protein; MR: mineralocorticoid receptor; NF-κB: nuclear factor k B; SNPs: single nucleotide polymorphisms; HDAC2: histone deacetylase 2; AP-1: activator protein 1. Adapted by permission from [Nature] [51].

The HPA axis regulation is highly complex. In addition to trans-synaptic regulation, the secretion of CRH by the PVN is also regulated by GCs, arginine vasopressin, and oxytocin [48]. The secretion of ACTH is regulated by GCs. The sensitivity of the adrenal cortex to ACTH is another possible limiting step in HPA axis activity. For in the serum, cortisol is bound to proteins—mainly corticosteroid-binding globulin (CBG)—where only 5% of systemic cortisol is free and bioactive (Figure 1) [49]. The availability of CBG, which is negatively regulated by GC, is another influence on GC actions. At the cellular level, cortisol availability is regulated by tissue-specific enzymes, as well as the 11β-hydroxysteroid dehydrogenases (11β-HSDs). 11βHSD-1 is widely expressed in all the GC target tissues (e.g., neurons and glial cells), where it converts cortisone to active cortisol, whereas 11βHSD-2 has the opposite effect, particularly in the kidney (Figure 1) [50]. At the cellular level, cortisol actions are also regulated by the GR and its respective *NR3C1* gene, as well as co-chaperones of the GR, such as FKBP5, among others.

Cortisol alone, therefore, cannot be considered to reflect the HPA axis regulation of the stress response. Furthermore, cortisol response to stressful events is moderated by sex—with women showing greater variability in the HPA axis response than men [52,53]. Testosterone has been shown to inhibit the stress response, whereas in women, estradiol has an important moderating role in the activation of the HPA axis in response to stress [7]. HPA axis stress response is also moderated by the stage of development and the age of the subject, as well as the type, intensity, and duration of the stressful event. Additionally, also important to mention is the fact that cortisol is difficult to measure accurately to represent the reflection of HPA axis activity, as most methods show considerable intraindividual variability and, as described above, HPA axis function depends on a great variety of factors [54].

### 2.2. The GC Receptors

The HPA axis—through the secretion of cortisol—is the primary hormonal mediator of stress responses (Box 1). GC actions are mediated by two types of receptors: the mineralocorticoid receptor (MR) and the GR. The MR has a 6–10-fold higher affinity for binding GCs than the GR [55]. The MR is basally occupied, while the GR is only activated during periods of stress and during the circadian peak of the HPA axis activity. The MR is important in terms of the time of day of HPA axis activity and the regulation of basal circadian and ultradian rhythms. Whereas the GR is widely found in the brain, the MR is restricted to the limbic areas [7]. The capability to cope with a TE depends on the balance of these two receptors in the regulation of the stress response [56] as low GC concentrations enhance MR homodimerisation, while GC stress levels induce MR–GR heterodimerisation [57]. The MR has a role in the appraisal of the threat and initiation of the response (Figure 1) [56].

The GR has three domains: the N-terminal transactivating domain (NTD), the DNA binding domain (DBD) (which binds to DNA GC response elements (GRE)), and the C-terminal ligand-binding domain (LBD) [58]. The NTD is the most variable domain, due to alternative translation initiation. There are many GR isoforms which depend on alternative splicing at several points of the *NR3C1* gene. Alternative translation initiation and post-translational modifications add to the functional pool of GC signalling diversity—which has tissue and cell specificity [59,60]. Epigenetic mechanisms which regulate gene transcription and expression have differential functional effects depending both on the gene’s region where they occur and on the SNPs’ makeup [61]. In addition, the location of the SNPs has specific influences on gene function [62]. 

The unbound GR is not alone in the cell’s cytoplasm, as it is associated with a multiprotein complex, including chaperone proteins and immunophilins [63]. Upon GC binding, this complex conformation changes and the GR becomes hyperphosphorylated and translocates to the nucleus, where it regulates the transcription of various genes directly and indirectly [46]. The GR binds to GRE as a homodimer directing transactivation of target genes. GC-induced gene expression is frequently cell type-specific, which has been shown to be dependent on the accessibility of the GR-binding site. In turn, this is determined by the chromatin structure and DNA methylation, as well as histone acetylation and methylation (HDAC2) (Figure 1) [58]. Bound-GRs also act as monomers, by interacting with other transcription factors, such as activator protein 1 (AP-1) and nuclear factor k (NF-kB), which represses their transcriptional activity and results in less pro-inflammatory effects (Figure 1) [64].

Furthermore, GCs have fast acting non-genomic actions which are mediated by cytosolic GRs and mGRs and use the activity of various kinases, thus adding greater complexity to GC signalling [65]. The effects are strongly dependent on both cell type and the cellular context. The existence of mGRs in the neuronal cells of the hypothalamus, basolateral amygdala, and hippocampus [42] plays an important role in rapid energy mobilisation and memory consolidation under stress, as well as rapidly preparing the cell for the ultimate genomic effects [66,67].

Chaperones and co-chaperones influence GR affinity for GCs. In particular, the co-chaperone of the heat shock protein (hsp)-90 *FKBP5* gene is subject to the transcriptional influence of the GR upon cortisol binding, leading to an increase in its expression, which is associated with GC resistance, serving as an intracellular feedback control of GC actions [68,69].

### 2.3. GR Gene and Other Genes that Regulate the HPA Axis

The most-studied gene related to the HPA axis is the GR *NR3C1* gene, which is located on chromosome 5q31-32 and is a member of the nuclear hormone receptor superfamily of ligand-activated transcription factors [70]. The human GR gene has nine exons, whereas exon 9 alternative splicing generates two GR isoforms: GRα and GRβ. The first exon is in effect a set of nine exons, which are referred to as A–J (excluding “G”), which are not translated and result in different mRNA, as they are independently controlled by a unique promoter which is located immediately upstream and thus influences the translation start site [71,72]. In the 5′ untranslated region of the *NR3C1* gene, there is a cytosine–phosphate–guanine dinucleotides (CpG) island which includes seven of the first exon’s set of nine exons: 1_D_, 1_J_, 1_E_, 1_B_, 1_F_, 1_C_ and 1_H_ [71] and is a target of DNA differential methylation studies. 

Several functional SNPs (Table 1) of the *NR3C1* gene have been extensively studied, namely: *TthIII*I, ER22/23EK, N363S, B*cl*I, and GR-9β [73].

The *TthIII*I SNP (rs10052957) is a C to T change in the promoter of the GR gene, and is only functional when associated with ER22/23EK [74].

The ER22/23EK polymorphism (rs6189/rs6190) is located in the transactivation domain and results in altered translation to the GR protein: glutamic acid-arginine (E-R) to glutamic acid-lysine (E-K) [75]. This SNP has been associated with relative GC resistance [76,77], which in turn has been attributed to the reduced transactivating capacity of the GR [78].

The N363S SNP (rs6195; alias rs56149945) results from one nucleotide substitution in codon 363 of exon 2, and the subsequent alteration from asparagine (N) to serine (S) [75]. This SNP is associated with increased sensitivity to GCs in vivo, which was manifested by significantly enhanced suppression of serum cortisol levels after a low dose of dexamethasone [79], which is probably due to increased transactivating capacity [10,76].

The *Bcl*I intronic SNP (rs41423247) which is situated downstream of exon 2, results from a C to G substitution at nucleotide 646, and has been associated with increased sensitivity to GCs [80,81]. This SNP was associated with emotional memory performance in healthy individuals [82], although its action mechanism is not known [10,73].

The 9β (also referred to as A3669G) SNP (rs6198) which consists of a naturally-occurring A to G substitution in the 3′ UTR of exon 9β [73], is associated with increased expression and stability of the GR*β* isoform of the GR [62], and with GC resistance, probably owing to decreased transrepression [10,83,84].

Other SNPs of the GR gene are associated with PTSD and depression, such as: rs258747 [18,85], rs10482612 [86], rs6191, rs33388 [87], rs6196, and rs10482605 [88].

The *FKBP5* gene, which is located in the human chromosome 6p21.31 [89], has also been extensively studied as it encodes a co-chaperone of hsp-90 in the GR molecular complex, which influences cortisol binding and subsequent conformational changes in the GR and the subsequent translocation to the nucleus [10]. FKBP5 is implicated in the feedback regulation of the GC response, as GCs increase *FKBP5* expression, which in turn decreases GR affinity for GCs. This is considered to be the ultra-short negative feedback loop for GR activity [90,91].

**Figure 2:**
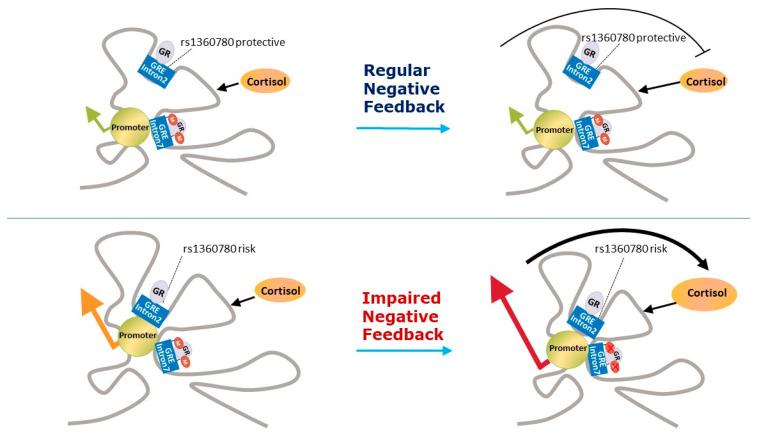
Representation of the epigenetic modification in *FKBP5*. The single-nucleotide polymorphism rs1360780 which is close to a functional GRE in intron 2 constitutes the genetic predisposition for an increased *FKBP5* transcriptional response to stress. Demethylation with higher cortisol levels is possibly restricted to certain developmental periods (e.g., childhood). The exposure to childhood trauma leads to an increased activation of *FKBP5* due to a reduction in DNA methylation in SNP risk allele carriers. Adapted by permission from [Nature] [94].

*FKBP5* overexpression is associated with GC resistance [68,69]. This increase in GC resistance has not been associated with higher plasma cortisol levels [92]. Four functional SNPs (Table 1) in the *FKBP5* gene (rs9296158, rs3800373, rs1360780, and rs9470080) have been identified which are associated with GR resistance, in normal, mainly Afro-descent individuals (Figure 2) [93].

The corticotrophin-releasing hormone receptor 1 (*CRHR1*) and 2 (*CRHR2*) genes have also been studied. In addition, variants for *CRHR1* (Table 1), rs110402, rs242924, rs7209436, rs12944712, rs12938031, and rs4792887 have been studied [93,95,96] and some of them demonstrate G × E interactions [97,98].

### 2.4. Epigenetic Regulation of the HPA Axis

Epigenetics refers to modifiable, potentially heritable, non-nucleotide changes to DNA transcription which are essential for normal cell differentiation and neurogenesis [99]. These changes can both be stable over time and dynamically responsive to environmental challenges such as nutritional, pharmacological, physical, and psychosocial changes, providing experience-dependent DNA modulation. Three main mechanisms have been identified for epigenetic gene transcription regulation and expression. The first is the DNA methylation of the cytosine pyrimidine ring of CpG sites, which suppresses gene transcription. It should be noted that these dinucleotides are especially numerous in the promoter regions (CpG islands). However, DNA methylation of the gene body can have the opposite effect [100]. The second mechanism consists of post-translational modifications of histone proteins, such as methylation, phosphorylation, and acetylation, which alter DNA that bind to regulatory proteins as well as the availability of chromatin for transcriptional activity. The third mechanism comprises RNAs (including siRNAs, miRNAs, and piRNAs) and long noncoding RNAs, which also regulate gene expression [99,101,102,103,104]. 

These modifications constitute the epigenome, which can record long-lasting influences from the environment and can be inherited [105,106]. These influences from the environment can occur at specific sensitive periods of development, including exposures in adulthood. DNA methylation is the most studied epigenetic mechanism of genes involved in HPA axis regulation, such as *NR3C1* and *FKBP5*. In particular, methylation of *NR3C1* is thought to underlie the programming of the HPA axis function in response to environmental exposures, such as childhood abuse [107,108]. Furthermore, higher cortisol levels demethylate *FKBP5* intron 7 in carriers of the SNP rs1360780 T risk allele possibly in specific developmental periods such as childhood, increasing *FKBP5* expression and GR resistance [94].

A review of the methylation effects on the GR gene concluded that early life adversity has been repeatedly shown to be associated with hypermethylation of the non-coding first exons [71], which could impair HPA axis functioning, specifically with the respective decreased GR expression, and predispose the exposed subjects to several psychiatric disorders.

The noncoding RNAs epigenetic mechanism has also been shown to influence the regulation of the HPA axis in response to stress. The miRNA miR-320a interacts with the *FKBP5* SNP rs3800373 C risk allele, leading to increased *FKBP5* expression and GR resistance [109]. Other studies of noncoding RNAs influences on the HPA axis regulation are reviewed elsewhere [110].

In sum, the HPA axis response to stress challenges has multiple levels of regulation which are influenced by variability in genes (SNPs) and epigenome. These effects can further expand environmental influences on genetic expression and consequently on endophenotypes and phenotypes [14]. This means that nature and nurture interactions have endless possibilities. This phenomenon can also be viewed from a therapeutic point-of-view, as these interactions can use pharmacotherapy and psychotherapy to environmentally manipulate these genes—by gene, by epigenome, and by environment interactions. 

## 3. HPA Axis and PTSD

As the new DSM-5 classification rightfully implies, PTSD is a disorder of the stress adaptation system, which is initiated by the influence of the TE on a susceptible person [111]. Accordingly, HPA axis dysfunction has been consistently pointed out to constitute the main pathophysiologic mechanism involved in the development and maintenance of PTSD [54,112,113]. 

Although PTSD only develops after exposure to TEs, other risk factors exist which increase the probability of a TE influencing the development of PTSD [4,6]. Some of these risk factors also influence the HPA axis function, examples being childhood adversity, parental PTSD, or the occurrence of prior TE or PTSD [9,28]. Exposure to these TEs generates anticipatory responses of the HPA axis, which in turn are moderated by SNPs and the epigenome [7,54,114]. Another important moderator between HPA axis functioning and PTSD is the biological sex, even when controlling for confounders. It is well known that women have a greater risk of developing PTSD than men [115,116]. Interestingly, female victims of sexual assault with lower hippocampal volumes showed increased risk of developing PTSD [117].

The most studied regulator of the HPA axis in PTSD is the GR [10]. Patients with PTSD show reduced GC signalling, which has been shown to be associated with increased GR responsiveness or sensitivity [113]. Indeed, hypersensitivity of the GR in PTSD patients has been found in several studies [111,118,119,120,121] and has been specifically attributed to decreased GC signalling [113]. In addition, studies which assess learning and memory also argue that PTSD patients have increased CNS GC hypersensitivity [122]. Indeed, cortisol plays an important role in coping with the emotional memories of trauma by enhancing consolidation and by decreasing retrieval and working memory [42].

Accordingly, lower cortisol levels in saliva, blood, or urine have been found in two metanalyses [118,123] in specific samples and assessment moments of the day, although lower cortisol levels were also found to be associated with trauma exposure, especially in the afternoon and evening. Another metanalysis, which included only saliva samples, showed that morning cortisol levels were significantly lower in PTSD patients when compared to controls [124]. Accordingly, a recent metanalysis found lower concentrations of 24-h urinary cortisol in PTSD patients when compared to controls [125]. Furthermore, neurobiological correlates of PTSD symptom clusters have also been found in some studies to show a negative association between cortisol levels and specific symptom clusters, such as intrusion, avoidance, and numbing e.g., [126,127].

On the other hand, CRH overactivity has been pointed out to be a possible model of HPA axis dysfunction in PTSD [54]. Central spinal fluid CRH levels have been found to be increased in patients with PTSD [128,129,130]. This finding is consistent with decreased GC signalling in PTSD, as GCs are potent inhibitors of CRH release [131]. Consistent with these HPA axis alterations is the concept that PTSD is a fear disorder, characterised by an excessively strong learning of trauma-related fear, or a failure over time to extinguish memories of previous trauma-related fear experiences [132]. 

Whether these HPA axis alterations are a cause or a consequence of PTSD development, or whether they are TE exposure-related has been a matter of debate in the scientific community [54]. HPA axis-related pre-trauma altered functions have been found to be more consistently associated with PTSD. High pre-deployment GR number, low *FKBP5* mRNA expression, and high glucocorticoid-induced leucine zipper (GILZ) gene mRNA expression are all independently associated with increased risk for a high level of PTSD symptoms six months after deployment to a combat zone [133]. Accordingly, lower hair cortisol concentrations prior to deployment were associated with post-deployment PTSD symptoms related to new stressors [134]. Furthermore, different clinical trajectories after experiencing a TE are also predicted by cortisol secretion as a response to experimental stress challenges, showing lower saliva cortisol change. When assessed prior to exposure to TEs, these trajectories are less-favourable on the whole [134,135]. With regards to peritraumatic HPA axis-related risk factors, lower cortisol levels [121,136] have been found to predict PTSD development, although a metanalysis only found that heart rate was correlated with posterior PTSD symptoms, among the other biological markers studied, including cortisol measured immediately after TE [137].

Imaging studies also link PTSD pathophysiology with the HPA axis. As an example, lower levels of expression of *FKBP5* were associated with smaller hippocampus and medial orbitofrontal cortex in PTSD patients when compared to non-PTSD controls [138]. A systematic review has shown that lower hippocampal volumes are associated with PTSD [139]. Excessive activation of the amygdala on exposure to threat stimuli has also been demonstrated in patients with PTSD [140].

### 3.1. PTSD and GC Regulation Genes SNPs

Genome wide association studies have failed to find associations between HPA axis-related genes and PTSD e.g., [141]. However, a recent meta-analysis found that SNPs in *NR3C1* and *FKBP5* genes (rs258747 in *NR3C1* and rs9296158 in *FKBP5*) are significantly associated with PTSD [18]. It has been hypothesised that some of the SNPs of the *NR3C1* gene associated with PTSD could be in linkage disequilibrium with previously-identified functional SNPs [85]. With regards *FKBP5*, the risk allele of the SNP rs9296158 has been previously associated with GR resistance in normal Afro-descent individuals and with GR hypersensitivity in PTSD patients (Figure 2) [93].

Although no main effects had been found for any of the previously-identified functional GR gene SNPs in PTSD, the severity of PTSD symptoms and basal plasma cortisol levels were negatively correlated within war veterans with PTSD who are homozygous for the B*cl*I SNP. In this same subgroup of PTSD patients, the authors also found a tendency for an increased response to a test of peripheral GC sensitivity which correlated with higher PTSD symptoms [142]. In cardiac surgery patients, homozygous carriers of the B*cl*I SNP variant allele showed significantly lower preoperative plasma cortisol levels and more traumatic memories six months after surgery and intensive care unit treatment [143]. A significant interaction effect was found of haplotype B*cl*I carrier state and childhood trauma on the pre-deployment GR number [133]. In this study, childhood trauma and a pre-deployment high GR number both predicted the subsequent development of a high level of PTSD symptoms. The higher GR number in the PTSD group was maintained after one and six months [10,144].

With regards *FKBP5*, the four functional SNPs which have been identified as being associated with GR resistance in normal, mainly Afro-descent individuals (rs9296158, rs3800373, rs1360780, and rs9470080) have all shown hypersensitivity to GC in patients with PTSD (Figure 2) [10,93]. This finding suggests that other factors influence the functionality of these SNPs. Interestingly, in this study, these SNPs significantly interacted with the severity of child abuse to predict levels of adult PTSD symptoms [93]. In a study on the genetics of substance dependence which also screened for PTSD, the *FKBP5* rs9470080 genotype (TT) was shown to moderate PTSD risk in interaction with childhood abuse in Afro–American participants, but not in the case of European–Americans [145]. Afro–Americans who were carriers of the TT genotype, but with no experience of childhood adversity, demonstrate the lowest risk for PTSD, but inversely they had the highest level if they had experienced childhood adversity. This result suggests that childhood adversity changes *FKBP5* gene functionality, as was the case in the study by Binder et al. [93]. A study of gene expression in 15 patients with PTSD, comparing them with 20 participants without PTSD, all of whom had been exposed to the World Trade Centre attacks, found that *FKBP5* expression was significantly reduced in current PTSD which was predicted by cortisol when entered with PTSD symptom severity in a regression analysis [146]. This study was extended to include SNP analyses of the *FKBP5* gene, as well as a sample of recovered PTSD patients and it was found that any of the four PTSD risk-related SNPs was a negative predictor of *FKBP5* expression, which in turn was related to lower levels of plasma cortisol and higher PTSD symptom severity [147]. Low pre-deployment levels of *FKBP5* mRNA expression were independently associated with the increased risk of high PTSD symptoms when assessed six months after deployment [148]. However, the study found no associations between *FKBP5* SNPs rs1360780 and rs3800373 haplotypes and pre-deployment *FKBP5* mRNA expression or GR number. On the other hand, in a sample of 412 chronic pain outpatients, the *FKBP5* gene SNP rs9470080 was associated with lifetime PTSD and it was found that participants without the risk allele had decreased PTSD risk, even in the presence of high levels of previous trauma exposure [149]. The interaction of genotype and PTSD symptoms associated with specific gene expression patterns suggested the existence of different biological PTSD endophenotypes, which are determined by functional SNPs in the *FKBP5* gene [150]. In this study, PTSD patients carrying the risk allele of the *FKBP5* gene SNP rs9296158 showed GR super-sensitivity with the dexamethasone suppression test, whereas those without the risk allele had lower baseline serum cortisol concentrations. PTSD carriers of this SNP risk allele had lower *FKBP5* mRNA expression, whilst those without PTSD had increased expression. However, in a study of 3890 US service members deployed to Iraq and Afghanistan, the carriers of the most frequent haplotype AGCC tended to have probable PTSD. Furthermore, each wild type variant SNP was associated with probable PTSD. In this study, the authors considered that *FKBP5* could be a risk factor for PTSD. No interactions with lifetime stressful events were found [151].

Klengel et al. [14] found that this diversity is determined by developmental impacts, such as early childhood adversities, which occurred by way of epigenetic mechanisms in the *FKBP5* SNP rs1360780 (Figure 2). This study showed that being a carrier of this *FKBP5* SNP risk allele, together with being exposed to early childhood adversities, both lead to *FKBP5* intron 7 demethylation. Furthermore, a recent meta-analysis found strong evidence of interactions between *FKBP5* genotypes in three SNPs (namely risk allele carriers of the rs1360780, rs3800373, or rs9470080) and early-life stress, which the authors considered could constitute significant risk factors for stress-related disorders such as PTSD [152]. Unfortunately, rs9296158 *FKBP5* SNP, which has been found to be associated with PTSD in a recent metanalysis [18], was not included.

The first study of the associations between *CRHR1* gene and PTSD in adults found that the SNPs rs12938031 and rs4792887 major alleles were associated with post-hurricane PTSD symptoms and that the former was also associated with PTSD diagnosis [96]. A longitudinal study of pediatric injury patients found an association of the *CRHR1* gene SNP rs12944712 with acute PTSD symptoms and their trajectory over time. Results from this study were considered preliminary, as the sample size was small [95]. One study identified that two SNPs (rs8192496 and rs2190242) in the *CRHR2* gene reduced PTSD risk among trauma-exposed female veterans. The minor allele of these SNPs was associated with reduced risk and severity of PTSD symptoms, although these findings have not been replicated [153]. 

The studies reviewed in this section show that GC regulation genes’ SNPs have an important role in PTSD risk and development. Studies have a greater focus on *FKBP5* gene variation, and on the whole, the literature shows that SNP alleles which are associated with HPA axis hypersensitivity are associated with PTSD. G × E interactions have also been shown, particularly in cases with experience of childhood adversity.

### 3.2. PTSD and Epigenetic Effects

The *NR3C1*, *FKBP5,* and *CRHR1* genes methylation have all been shown to be associated with childhood adversity [8,14,108,154]. These modifications could constitute risk factors for PTSD development upon later exposure to a TE. Epigenetic modifications could also result from TE proper exposure and influence the pathophysiology of PTSD development. Methylation levels of *NR3C1* and *FKBP5* have been repeatedly associated with the status of PTSD diagnosis [155].

Despite the different methodologies across studies, a recent systematic review found strong evidence of an association between *NR3C1* increased methylation levels and decreased gene expression, which suggests a role for this gene’s methylation in stress-related psychopathology [156]. Methylation levels of the *NR3C1* gene have been studied in the promoter regions of exons 1_B_, 1_C_, and 1_F_, which are rich CpG sites. Hypermethylation of 1_F_ is associated with a decreased expression of the gene and accordingly with GR resistance [108]. An association between the demethylation of the GR gene exon 1_F_ promoter and both PTSD and plasma cortisol decline on the dexamethasone suppression test was found [157]. In this study, war-related PTSD male subjects showed greater cortisol suppression after the administration of dexamethasone, and higher levels of peripheral blood mononuclear cell lysozyme inhibition in the lysozyme suppression test. Accordingly, another study of survivors of the Rwanda genocide found that the sex-dependent salivary GR gene exon 1_F_ promotor DNA methylation was associated with PTSD [158]. Male but not female survivors with increased GR gene exon 1_F_ promotor methylation, which was associated with lower *NR3C1* expression, showed less intrusive memory of the traumatic event and reduced PTSD risk. In these studies, it was not possible to distinguish the effects of trauma from the effects of PTSD. Another study focussing on female victims of the same Rwanda Tutsi genocide, found higher peripheral blood leukocytes *NR3C1* exon 1_F_ promotor methylation levels than those of Tutsi non-victims [159]. However, the association between PTSD and *NR3C1* gene exon 1_F_ promotor methylation levels was not addressed, and in this case, hypermethylation could be attributed to trauma exposure. *NR3C1* exon 1_F_ promotor methylation levels negatively correlated both with cortisol levels and *NR3C1* mRNA expression levels. In this same study, those exposed to trauma showed higher methylation of CpGs located within the *NR3C2* coding sequence than non-exposed subjects. On the other hand, peripheral T lymphocytes *NR3C1* 1_B_ and 1_C_ promoters’ methylation levels were found to be lower in subjects with lifetime PTSD related to different types of trauma, when compared with non-traumatised controls. Cortisol levels were inversely correlated with *NR3C1* 1_B_ mRNA expression. Furthermore, overall and CpG site-specific methylation levels were inversely correlated with total *NR3C1* and 1_B_ mRNA expression [160]. However, in this study, the *NR3C1* exon 1_F_ promotor methylation did not associate with PTSD risk. Another study of female interpersonal violence victims found PTSD severity to be negatively correlated with the mean percentage of *NR3C1* exon 1_F_ methylation [161].

*FKBP5* methylation has also been extensively studied. Klengel et al. [14] found that being a carrier of the *FKBP5* SNP rs1360780 risk allele T and also being exposed to early life trauma lead to *FKBP5* intron 7 demethylation. *FKBP5* intron 7 is situated in a GC response element zone, which is subject to the action of GCs as part of the ultra-short feedback loop between FKBP5 and the GR, which leads to GR resistance, as demethylation increases levels of this co-chaperone. However, [162] no main effects of PTSD diagnosis on *FKBP5* intron 7 methylation were found in a sample of Holocaust survivors, nor in their offspring and comparison subjects. A recent study did find significantly higher intron 7 methylation levels among veterans with PTSD carrying the rs1360780 risk allele when they were compared to the non-PTSD group [163]. On the other hand, *FKBP5* exon 1 promoter methylation was associated with lower plasma cortisol levels in subjects with combat-related PTSD [33], which supports previous findings of lower *FKBP5* expression in PTSD [146].

In the case of GILZ—which is a transcription factor that is up-regulated by GC action [46]—mRNA levels were associated with PTSD in males, and were negatively associated with the methylation of the respective gene. Furthermore, the number of TEs correlated negatively with *GILZ* mRNA levels, and positively with the percentage methylation of *GILZ* just in the case of males [164].

Other approaches to the epigenomic study of PTSD pathophysiology are still being carried out. A longitudinal study of combat-related PTSD found decreases in DNA methylation in three novel genomic regions *(ZFP57*, *RNF39* and *HIST1H2APS2*) across the period of exposure which constituted marks of susceptibility to PTSD [165]. Noncoding RNAs have also been implicated in the relationship between stress and GCs and these could prove to be useful biomarkers to facilitate the prescription of personalised medicines for trauma-related disorders, as the majority of PTSD blood based microRNA studies report reduced expression in PTSD [110]. 

Differences in gene expression can reflect epigenetic modifications and/or interactions with SNPs. Changes in gene expression have been found to occur after combat exposure [166]. However, no longitudinal studies have examined the combination of DNA methylation and gene expression—which limits our understanding of associated TE exposure DNA methylation on gene expression [166].

The epigenetic regulation of gene expression is very much cell-type specific [112]. The previous studies researched methylation in non-CNS cells, and therefore we are unable to conclude on the neuronal methylation status, although this could well reflect exposure to environmental influences [100], as has been shown for psychosocial adversity [167] and combat [165], which accordingly could represent biomarkers of the brain related phenotype. In addition, substantial correlations between blood and brain samples have been observed in post-mortem studies [168]. On the other hand, the epigenetic modifications could be the result of PTSD effects on the HPA axis and immune system, as DNA methylation changes in response to environmental exposure can be induced by altered GC signalling [169].

There are also reports of intergenerational transmission of these epigenetic modifications [105,162], which could constitute one of the many mechanisms for intergenerational transmission of PTSD [28]. 

In brief, PTSD has been consistently associated with lower levels of cortisol and GR hypersensitivity. Furthermore, a meta-analysis found associations between variability in the *NR3C1* and *FKBP5* genes and PTSD. Other SNPs in genes involved in HPA axis regulation have also been found to be associated with PTSD, such as the *CRHR1.* Epigenome studies have found evidence of associations between levels of methylation in several genes with a regulatory role in HPA axis functioning—mainly *NR3C1* and *FKBP5*—which can depend on the gene’s SNPs and on the gene’s location of methylation. These findings are consistent with previous evidence of lower cortisol levels and GR hypersensitivity in PTSD. All this genetic makeup variability has been found to interact with environmental influences, consubstantiating a new paradigm of gene × trauma × epigenetic interactions [155].

## 4. PTSD Treatments Which Influence the HPA Axis

### 4.1. GC Treatments

PTSD treatments using GR as a mediator have been tested for use as secondary prevention, that is to say, on exposure to a TE [54]. Based on studies which show that low peritraumatic cortisol constitutes a risk factor for PTSD development, GCs could be of benefit if administered immediately after the TE [170,171,172,173], especially in the case of individuals with this additional risk factor. A large randomised control study found that traumatic injury patients who received hydrocortisone within 12 h after trauma for 10 days reported fewer PTSD symptoms during the first three months post trauma than did patients who received a placebo [174]. On the other hand, GC antagonists could also be useful, based on the role of GCs in the consolidation of the traumatic memories [42]. As GC actions influence so many functions in the brain [46], the development of synthetic GCs or other GC action modulators with increased tissue/cell/molecular pathway selectivity could be of great benefit for patients, as they would act on the specific PTSD HPA axis-related pathophysiological mechanism [54,112]. However, research is still far from finding such “chirurgical” treatments.

Several clinical trials are currently being carried out with substances which aim to influence the HPA axis in PTSD patients [112]. These trials are supported by studies such as that by Aerni et al. [175]—a pilot study which found that a low dose of hydrocortisone administered over three months reduce re-experiencing and avoidance symptoms in PTSD patients, based on the assumption that elevated cortisol levels which inhibit memory retrieval in healthy human subjects can also reduce the excessive retrieval of traumatic memories and related symptoms in patients with chronic PTSD. Unfortunately, other larger placebo-controlled randomised trials targeting several HPA axis-related PTSD pathophysiological mechanisms did not achieve the same promising results [176,177,178].

Furthermore, hydrocortisone supplementation of prolonged exposure therapy for PTSD in military veterans has been associated with a lower dropout rate and greater reduction in total PTSD symptoms, when compared to a placebo [179]. Responders to hydrocortisone supplementation had the highest pre-treatment GC sensitivity, which decreased as symptoms improved.

Because *FKBP5* SNPs, methylation, and expression variation have all been consistently associated with PTSD pathophysiology, *FKBP5* is a potential treatment target, as it confers risk for abnormal fear extinction learning in humans [180].

A recent study by Pape et al. [181], which investigated the CRHR1 antagonist GSK561679 in female PTSD patients supports the possible role of *CRHR1* methylation levels as an epigenetic biomarker to follow response to CRHR1 antagonist treatment in specific subgroups. Moreover, pre-treatment *NR3C1* methylation levels may be useful as a potential biomarker to predict PTSD treatment outcome [33].

### 4.2. Psychotherapy for PTSD

Several psychotherapeutic interventions have been indicated for PTSD treatment. Some practice guidelines recommend them as first-line treatment [112,182,183]. Psychotherapeutic interventions for PTSD include both trauma-focussed ones, which are based on processing the emotional and cognitive aspects of the traumatic event, and non-trauma-focussed ones. Trauma-focussed approaches include prolonged exposure therapy (PE), cognitive processing therapy (CPT), and eye movement desensitisation and reprocessing (EMDR), which have all been considered first line psychotherapies [29,112]. On the other hand, stress inoculation training has been considered as first line non-trauma-focused psychotherapy for PTSD patients [29]. Recently, the guidelines are being increasingly questioned, as PE and CPT have high dropout rates, and only a minority of patients cease to be diagnosed with PTSD at the end of treatment [30,31]. Furthermore, recent trials have found no differences between several therapies, including the administration of sertraline hydrochloride and non-trauma-focussed therapies which were not previously considered to constitute first line psychotherapies for PTSD patients [31]. This highlights the importance of understanding the complexity and diversity of PTSD in each patient and also the need for the clinician to be able to adapt to the patient’s needs, which can change over time [30]. Interestingly, Steenkamp et al. [31] state the need for long-term personalised approaches which can rely on the building of a therapeutic relationship to achieve better outcomes. Psychodynamic psychotherapies are also useful for the treatment of PTSD [184] and quality-based reviews of randomised controlled trials have shown no significant difference when compared with other psychotherapies, such as cognitive-behavioural therapy [185,186].

Psychotherapeutic interventions differ in a number of characteristics, such as the theoretical foundations, objectives, frequency of sessions, duration, required training of therapists, and the personal conditions required of potential candidates [187]. Although 30% of the results of psychotherapy are attributed to factors which are common to all of them [188], ideally we should use which ever psychotherapeutic intervention better works for each patient [189]. 

Daskalakis et al. [190] proposed a tree-hit model of the individual’s programming sensitivity to environmental stress, depending on the timing of environmental exposure—should this have occurred during highly plastic developmental phases [191]. The genetic makeup (hit-1) interacts with early-life environmental exposure (hit-2), resulting in (endo) phenotypes (e.g., epigenetic changes and altered HPA axis function) [14] which constitute vulnerability or resilience factors, depending on the type and characteristics of later-life environmental challenges (hit-3) which confront the individual [190]. These later-life environmental challenges can also constitute the basis for psychotherapeutic repair when this three-hit model results in vulnerability and psychic suffering. 

#### 4.2.1. Psychotherapy and HPA Axis 

PTSD is considered to be a memory-related fear disorder, which is characterised by an over-consolidation of TE-related fear, or, on the other hand, a failure to extinguish fear memories [54]. GCs have potentiating effects on emotional memories consolidation and impair memory retrieval. Several theories exist as to which mechanisms are involved in PTSD memory-related pathophysiology [54], including over-consolidation due to excessive noradrenergic signalling as a consequence of low levels of GCs [192] or due to GC-enhanced (as a result of GR hypersensitivity) fear memory consolidation [42]. After consolidation, memories can be retrieved. Important consequences after retrieval are the reconsolidation and extinction of memories, which seem to share the same role of GCs in both the amygdala and hippocampus as that of consolidation [54,193]. This is an important factor, as some PTSD psychotherapeutic interventions rely on fear learning paradigms which can be enhanced by GCs, depending on the knowledge of the adequate moment, dose, and choice of the GC to be administered. On the other hand, a GR antagonist might be more adequate if our aim is the GR hypersensitivity pathophysiological hypothesis of PTSD [54]. One example of this is the case of the most studied psychotherapy indicated for treatment of PTSD, that of PE, which relies on fear learning paradigms, particularly extinction learning, which, in turn, depend largely on the actions of GCs [54]. Extinction includes repeated presentations of the feared stimulus without reinforcement. Interindividual variability in extinction learning can be mediated in part by SNPs in *FKBP5* [180] and constitute one of the reasons for different treatment responses in patients with PTSD. 

The first study to address the association of HPA axis-related risk factors with psychotherapeutic outcomes in PTSD patients showed that after brief eclectic psychotherapy, significant changes occurred in levels of cortisol and dehydroepiandrosterone in PTSD patients with civilian-related trauma. Those who responded to therapy showed an increase in cortisol and dehydroepiandrosterone levels, while for those who did not respond, both hormone levels decreased [32].

Accordingly, a higher bedtime salivary cortisol and 24-h urinary cortisol excretion predicted positive treatment outcomes in PTSD veterans after PE therapy or a weekly minimal attention intervention for 12 consecutive weeks [35]. Another study of combat-exposed male PTSD patients found that a high cortisol awakening response (CAR_i_) before trauma-focussed psychotherapy predicted symptom reduction after 6–8 months. In this study, no differences were found between the PTSD and the non-PTSD group who had received no treatment with regard to a decrease in CAR_i_ at the end of the treatment period [194]. Furthermore, a study of veterans with PTSD showed that an increased salivary cortisol response to personal trauma script-driven imagery task prior to PTSD therapy was significantly and uniquely related to reductions in the core symptoms of PTSD after PE [195]. Interestingly, an 8-week psychosocial intervention with war-affected adolescents regulated hair cortisol levels. While this intervention decreased hair cortisol levels for young patients with hypersecretion and medium secretion, it increased hair cortisol levels for young patients with hyposecretion, in relation to the control group [196]. Steudte-Schmiedgen et al. [197] suggest that hair cortisol levels can be valuable to complement research into long-term HPA axis predictors and correlates of clinical outcomes in response to psychotherapy for PTSD patients. 

A literature review revealed preliminary evidence that the lower levels of pre-treatment GC are related to poorer PTSD psychotherapeutic treatment gains [12]. The authors hypothesised that HPA axis dysregulation increases both behavioural and emotional avoidance, which interferes with the efficacy of psychotherapy, especially exposure-based interventions. This could constitute a strong argument for GC-enhanced psychotherapy. Another argument could be related to the fact that GCs can potentiate extinction learning [180] or decrease the retrieval of TEs memories on exposure to stimuli [198], either spontaneously [199], or during the application of PE therapy [179]. 

Imaging studies have also been carried out to understand whether structural modifications occur after psychotherapy. A metanalysis revealed that psychotherapy effects in PTSD increased prefrontal and decreased limbic activity, particularly by normalising hippocampal function and morphology [200]. Later studies continued to confirm these findings. Butler et al. [201] found hippocampal grey matter increases after six weeks of multimodal psychological treatment for combat-related PTSD, when compared with a waiting-list control group. Another study also found that clinical improvement during cognitive behavioural therapy in PTSD was predicted by increased *FKBP5* expression and increased hippocampal size [138]. Increased hippocampal volume and elevated *FKBP5* expression were significantly correlated. The hippocampus has been shown to be highly sensitive to the effects of GCs [202]. These studies highlight the key role that hippocampal neuroplasticity can play in improving resilience and recovery from traumatic stress, especially bearing in mind its involvement in the HPA axis regulation of memory extinction [54] and also that it is one of the major structures of neurogenesis in the adult human brain [203]. 

#### 4.2.2. Psychotherapy Interaction with Genetic Makeup in PTSD 

Since G × E interactions are determined by a certain confluence of environmental factors on SNPs and epigenomic mechanisms, and as these interactions exert an influence on the HPA axis-related pathophysiology of PTSD development and maintenance, we can accordingly presume that G × E interactions could be beneficial for the treatment of these and other disorders—if we could determine the terms of the interaction. In other words, what environmental change would be required for which genetic makeup? This environmental change could be a psychotherapeutic intervention. 

Psychotherapeutic effects result from learning and memory-related neuronal plasticity [204] which ultimately depend on intervention-associated gene expression [205]). As SNPs and epigenetic changes have direct effects on gene expression, several lines of investigation have tried to better understand these relationships. 

HPA axis-related genetic makeup information can be useful in psychotherapy practice through several of the following ways. Firstly, the SNPs of genes regulating the HPA axis function, such as *NR3C1*, *FKBP5*, and *CRHR1*, can interact with psychotherapeutic interventions in patients with specific PTSD characteristics and result in better outcomes. This knowledge can enable informed choices of the best intervention. Likewise, knowledge of the epigenome that is associated with better outcomes can also inform the decision regarding the most appropriate psychotherapy. This information could be constituted by biomarkers for both the indication and outcome of the psychotherapies for specific patients, as, for example, appears to happen in the case of a high level of pre-treatment cortisol as a predictor of better treatment outcomes [35,194,195]. In addition, psychotherapy addresses the trauma which seems to be the main culprit in HPA axis dysregulation interacting with genes.

Furthermore, the usefulness of genetic makeup information for the prescription of psychotherapy and the respective outcomes is promising, based on the results of several studies which have researched the interaction between psychotherapy and both SNPs and the epigenome.

The B*cl*I SNP G carrier states predicted treatment gains after 12 consecutive weeks with PE therapy, or a weekly minimal attention intervention for patients with PTSD [35]. Homozygotes of the same B*cl*I SNP risk allele evidenced more traumatic memories of intensive care unit treatment at six months after cardiac surgery [143]. Taken together, this information could help to identify those subjects who are at higher risk of PTSD symptoms and provide them with a psychotherapeutic intervention before, or shortly after the TE exposure, depending on its predictability. Interestingly, a randomised controlled trial study showed that a pre-operation minimal cognitive behavioural intervention which targeted homozygous carriers of this SNP’s G high-risk allele reduced traumatic memories and posttraumatic stress disorder symptoms after heart surgery [206]. Interestingly, GG carriers of this SNP showed increased emotional memory performance when compared to GC and CC carriers [82].

Traumatised Ugandan carriers of the *FKBP5* rs1360780 risk allele were at increased risk of PTSD symptoms relapse after 10 months of Narrative Exposure Therapy (a form of exposure-based short-term therapy), whereas non-carriers continued to show a reduction in symptoms [36]. This is an interesting result, as the genetic makeup which correlates with functional distinct roles impacts on different treatment outcomes [14,152].

Gene expression studies have also shown that increased *FKBP5* expression after treatment is associated with successful response to trauma-focussed psychotherapy [33,138]. Levy-Gigi et al. [138] found significant increases in *FKBP5* expression and in hippocampal size, which were correlated, in PTSD patients after 12 weekly 1.5-h sessions of trauma-focussed cognitive behavioural therapy. Improvement in PTSD symptoms was predicted by increased *FKBP5* expression and increased hippocampal volume, although the primary predictor was *FKBP5* expression [138].

A recent review of studies addressing the associations between various psychiatric disorders and several genes methylation before and after psychotherapeutic interventions concluded that DNA methylation change can be considered a marker of treatment outcome [100]. However, the author advises adopting caution in relation to the interpretation of results, owing to different methodologies across studies and also the need for the biological functional characterisation of the findings.

Higher *NR3C1* exon 1_F_ promoter methylation levels predict a better treatment response after 12 weeks of PE psychotherapy, whereas *FKBP5* exon 1 promoter region methylation levels decreased in those subjects who responded with remission to the same psychotherapy. Correspondingly, those subjects who responded to treatment had higher *FKPP5* gene expression than those who did not respond [33]. The authors argue that GR gene methylation did not change in response to treatment, due to association with early environmental changes which could be more enduring. The methylation changes could constitute biomarkers of PTSD prognosis and symptom severity, respectively, as the environment part of G × E interactions could be attributed to epigenetic mechanisms [14].

Other studies addressing epigenomic mechanism in other genes associated with PTSD psychotherapy are currently being carried out. A longitudinal study of genome-wide DNA methylation levels found that the successful treatment of PTSD patients with trauma-focused psychotherapy (cognitive behavioural therapy, with or without EMDR) was accompanied by changes in DNA methylation at 12 differentially methylated genomic regions and particularly of the *ZFP57* gene with regards to prospective evidence [34,207]. *ZFP57* codes for a transcriptional regulator of genomic imprinting which has been associated with hippocampus-related stress vulnerability [208]. 

The previous research reviewed shows that the HPA axis is an important mediator of treatment approaches to PTSD, based on the fear learning paradigm of PTSD pathophysiology. Although promising, pharmacotherapy targeting the HPA axis still has a long way to go. In addition, GC-enhanced psychotherapy also represents a possible approach which needs to be investigated better. Indeed, psychotherapy research for treating PTSD needs to be pursued, as outcomes are far from good. It seems that certain endophenotypes can be useful in predicting treatment outcomes and psychotherapy has shown to normalise HPA axis-related functions in several ways, including levels or cortisol, gene expression, and CNS function and structure. The way genetic makeup (namely SNPs and DNA methylation) interacts with psychotherapy can also be useful to inform us of the best treatment options. However, further studies of these relationships are needed before we can apply these interesting discoveries to clinical practice.

## 5. Conclusions

### 5.1. Clinical Implications

**Figure 3:**
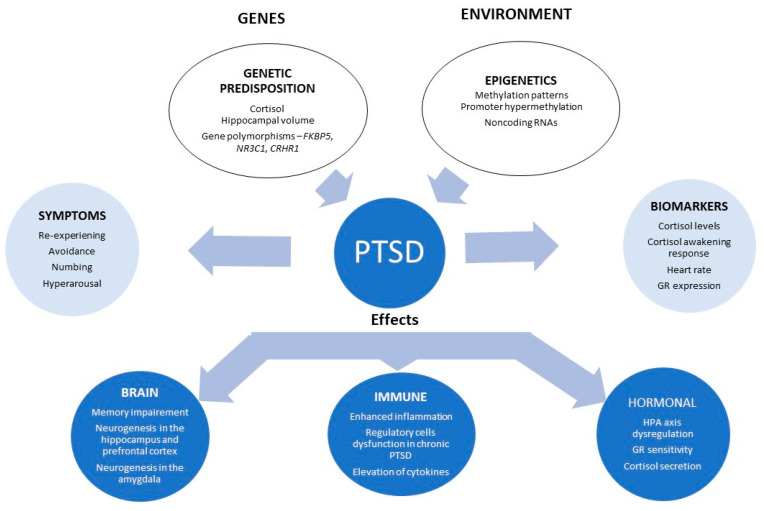
Gene/environment interaction in PTSD. Ranging from genetic and environment interactions through to symptoms, possible biomarkers and brain and hormonal changes. Abbreviations: HPA axis: hypothalamic–pituitary–adrenal axis; GR: glucocorticoid receptor. Adapted from [209].

PTSD is associated with many vulnerability and risk factors [6]. Several pathophysiological mechanisms have been hypothesised [54], which make this disorder phenotypically so diverse [19], and thus, treatments must inevitably be tailored, as we are now considering this disorder to be a gene–trauma–epigenetic interactions paradigm (Figure 3) [155]. 

Evidence is mounting that the HPA axis is implicated in PTSD pathophysiology and that interventions aiming to affect this system are showing increased usefulness for treatment interventions and can inform treatment outcomes—particularly psychotherapeutic interventions [e.g., 33,138,194].

We hope to have made it clear that traumatic experiences interact with the genetic makeup in several ways during human development. One of these is through modifications of the epigenome conditioning of the pathophysiology of many disorders—including PTSD. However, probably the most important message is that this interaction can occur reversely, through psychotherapeutic interventions which, if tailored according to the pathophysiological mechanisms, could be more useful for those individuals who suffer, and more cost-effective for all [33], although further studies need to be carried out to inform us better what works best, and for whom. If we could predict which patients would improve better with which type of treatment, then the level of suffering, time, and resources could be significantly reduced for such a complex and heterogeneous disorder. However, as promising and appealing the results of the studies reviewed in this paper might be, there is still a long way before they attain clinical applicability.

Furthermore, psychotherapy delivered perinatally with modifications which are specific to the perinatal period and are focussed on parenting are necessary for the prevention of PTSD intergenerational transmission [210], as PTSD influenced parent-child interactions are important and potentially modifiable contributors [211]. Evidence-based psychotherapy offers considerable advantages for perinatal PTSD, based on the potential teratogenic effects of typically-recommended medications for PTSD [212]. Furthermore, epigenetic modifications associated with PTSD are subject to inheritance [105]. If these epigenetic modifications could be reversed by psychotherapy, this would then constitute another way to break the intergenerational transmission of PTSD.

The studies mentioned in this review used psychotherapy for PTSD secondary or tertiary prevention or treatment, however, as it is known that there is a genetic risk for exposure to TEs, a primary psychotherapeutic intervention might also be useful, mainly for those individuals with other cumulative risk factors, such as childhood adversity or parental psychiatric disorders [6]. Furthermore, this review can also be useful to support the importance of trauma-informed care as a patient-centred approach which is needed in clinical practice and which can prevent many forms of trauma-related suffering [213]. 

### 5.2. Future Directions

Psychiatric disorders are multi-determined and have a complex pathophysiology and multiple phenotypic expressions, and therefore any information which could help tailor treatments should be pursued—in particular longitudinal gene x psychotherapy interaction studies. 

In the case of PTSD, most of the psychotherapeutic interventions that have been studied are short-term, limited interventions. It would be interesting to study the effects of intensive interventions, which require treatments of more than once a week, with no time limits (e.g., psychoanalysis—which implies 3–5 sessions a week, also with no time limit). These interventions would address not only Daskalakis et al.’s [190] hit-3 environmental challenges, but also hit-2 early-life environmental exposure. What would be the changes in HPA axis biomarkers (endophenotypes) and the epigenetic changes? Would an association or interaction with SNPs of genes regulating HPA axis be found? Further research regarding the interactions between psychotherapy and the epigenome are warranted.

Studies are needed which focus on genetic makeup and their functional correlates, which can identify endophenotypes that constitute relevant mediators or moderators of treatment efficacy. A limited number of studies examine mRNA expression in relation to epigenetic modifications in patients with PTSD. This research is important, in order to find functional correlates between methylation patterns and phenotypic variability [71]. A better selection of the region to study DNA methylation also needs to be pursued, as well as other epigenetic mechanisms and their functional correlates [71,110]. In addition, larger samples should be studied to increase the study’s power with randomised control trial approaches. Genome-wide studies of therapy responses would be ideal.

In the future, it should be possible to integrate different biomarkers—including SNPs, the epigenome, and gene expression profiles—with clinical variability and psychotherapeutic armamentarium with the objective to better inform those clinicians who practice personalised/patient-centred care for this complex and challenging disorder of PTSD.

## Figures and Tables

**Table 1:** Single nucleotide polymorphisms (SNPs) of the *NR3C1*, *FKBP5*, and *CRHR1* genes which have been associated with post-traumatic stress disorder (PTSD) or which have altered sensitivity of the hypothalamus–pituitary–adrenal axis.

Gene	SNP Custom Name	dbSNP ID	Wild Allele	Variant Allele	GR Sensitivity *
*NR3C1*	*TthIII*I	rs10052957	C	T	↓ ^†^
	ER22/23EK	rs6189/rs6190	G/G	A/A	↓
	N363S	rs6195 (rs56149945) ^‡^	A	G	↑
	*Bcl*I	rs41423247	C	G	↑
	GR-9β (A3669G) ^¥^	rs6198	A	G	↓
		rs258747	A	G	?
		rs10482612	G	A	↓
		rs6191	C	A	↓
	NR3C1-1	rs10482605	T	C	↓
					
*FKBP5*		rs3800373	A	C	↓
		rs9296158	G	A	↓
		rs1360780	C	T	↓
		rs9470080	C	T	↓
					
*CRHR1*		rs110402	G	A	↓
		rs242924	G	T	?
		rs12944712	G	A	?
		rs12938031	A	G	?
		rs4792887	C	T	?

* Studied specifically for GR sensitivity or inferred from functional research. ^†^ Only with the ER22/23EK; ↑: GR hypersensitivity; ↓: GR resistance; ?: unknown. ^‡^ Aliases. ^¥^ Also known as. Abbreviation: dbSNP ID: Single Nucleotide Polymorphism Database identification. Adapted by permission from [10].

## References

[1] American Psychiatric Association (2013). Diagnostic and Statistical Manual of Mental Disorders.

[2] Breslau N., Kessler R.C., Chilcoat H.D., Schultz L.R., Davis G.C., Andreski P. (1998). Trauma and posttraumatic stress disorder in the community: The 1996 Detroit Area Survey of Trauma. Arch. Gen. Psychiatry.

[3] Creamer M., Burgess P., McFarlane A.C. (2001). Post-traumatic stress disorder: Findings from the Australian National Survey of Mental Health and Well-being. Psychol. Med..

[4] de Vries G.J., Olff M. (2009). The lifetime prevalence of traumatic events and posttraumatic stress disorder in the Netherlands. J. Trauma. Stress.

[5] Naifeh J.A., North T.C., Davis J.L., Reyes G., Logan C.A., Elhai J.D. (2008). Clinical profile differences between PTSD-diagnosed military veterans and crime victims. J. Trauma Dissociation Off. J. Int. Soc. Study Dissociation.

[6] Xue C., Ge Y., Tang B., Liu Y., Kang P., Wang M., Zhang L. (2015). A meta-analysis of risk factors for combat-related PTSD among military personnel and veterans. PLoS ONE.

[7] Herman J.P., McKlveen J.M., Ghosal S., Kopp B., Wulsin A., Makinson R., Scheimann J., Myers B. (2016). Regulation of the Hypothalamic-Pituitary-Adrenocortical Stress Response. Compr. Physiol..

[8] Ramo-Fernandez L., Boeck C., Koenig A.M., Schury K., Binder E.B., Gundel H., Fegert J.M., Karabatsiakis A., Kolassa I.T. (2019). The effects of childhood maltreatment on epigenetic regulation of stress-response associated genes: An intergenerational approach. Sci. Rep..

[9] Khoury J.E., Bosquet Enlow M., Plamondon A., Lyons-Ruth K. (2019). The association between adversity and hair cortisol levels in humans: A meta-analysis. Psychoneuroendocrinology.

[10] Castro-Vale I., van Rossum E.F., Machado J.C., Mota-Cardoso R., Carvalho D. (2016). Genetics of glucocorticoid regulation and posttraumatic stress disorder--What do we know?. Neurosci. Biobehav. Rev..

[11] Hawn S.E., Sheerin C.M., Lind M.J., Hicks T.A., Marraccini M.E., Bountress K., Bacanu S.A., Nugent N.R., Amstadter A.B. (2019). GxE effects of FKBP5 and traumatic life events on PTSD: A meta-analysis. J. Affect. Disord..

[12] Colvonen P.J., Glassman L.H., Crocker L.D., Buttner M.M., Orff H., Schiehser D.M., Norman S.B., Afari N. (2017). Pretreatment biomarkers predicting PTSD psychotherapy outcomes: A systematic review. Neurosci. Biobehav. Rev..

[13] Fernandez C.A., Choi K.W., Marshall B.D.L., Vicente B., Saldivia S., Kohn R., Koenen K.C., Arheart K.L., Buka S.L. (2020). Assessing the relationship between psychosocial stressors and psychiatric resilience among Chilean disaster survivors. Br. J. Psychiatry.

[14] Klengel T., Mehta D., Anacker C., Rex-Haffner M., Pruessner J.C., Pariante C.M., Pace T.W., Mercer K.B., Mayberg H.S., Bradley B. (2013). Allele-specific FKBP5 DNA demethylation mediates gene-childhood trauma interactions. Nat. Neurosci..

[15] Mehta D., Miller O., Bruenig D., David G., Shakespeare-Finch J. (2020). A Systematic Review of DNA Methylation and Gene Expression Studies in Posttraumatic Stress Disorder, Posttraumatic Growth, and Resilience. J. Trauma Stress.

[16] Sartor C.E., Grant J.D., Lynskey M.T., McCutcheon V.V., Waldron M., Statham D.J., Bucholz K.K., Madden P.A., Heath A.C., Martin N.G. (2012). Common heritable contributions to low-risk trauma, high-risk trauma, posttraumatic stress disorder, and major depression. Arch. Gen. Psychiatry.

[17] True W.R., Rice J., Eisen S.A., Heath A.C., Goldberg J., Lyons M.J., Nowak J. (1993). A twin study of genetic and environmental contributions to liability for posttraumatic stress symptoms. Arch. Gen. Psychiatry.

[18] Sheerin C.M., Lind M.J., Bountress K.E., Marraccini M.E., Amstadter A.B., Bacanu S.A., Nugent N.R. (2020). Meta-analysis of Associations Between Hypothalamic-Pituitary-Adrenal Axis Genes and Risk of Posttraumatic Stress Disorder. J. Trauma Stress.

[19] Galatzer-Levy I.R., Bryant R.A. (2013). 636,120 Ways to Have Posttraumatic Stress Disorder. Perspect. Psychol. Sci..

[20] Bonanno G.A., Mancini A.D., Horton J.L., Powell T.M., Leardmann C.A., Boyko E.J., Wells T.S., Hooper T.I., Gackstetter G.D., Smith T.C. (2012). Trajectories of trauma symptoms and resilience in deployed U.S. military service members: Prospective cohort study. Br. J. Psychiatry.

[21] Bryant R.A., Nickerson A., Creamer M., O’Donnell M., Forbes D., Galatzer-Levy I., McFarlane A.C., Silove D. (2015). Trajectory of post-traumatic stress following traumatic injury: 6-year follow-up. Br. J. Psychiatry.

[22] Galatzer-Levy I.R., Madan A., Neylan T.C., Henn-Haase C., Marmar C.R. (2011). Peritraumatic and trait dissociation differentiate police officers with resilient versus symptomatic trajectories of posttraumatic stress symptoms. J. Trauma Stress.

[23] Pietrzak R.H., Van Ness P.H., Fried T.R., Galea S., Norris F.H. (2013). Trajectories of posttraumatic stress symptomatology in older persons affected by a large-magnitude disaster. J. Psychiatr. Res..

[24] Kehle S.M., Reddy M.K., Ferrier-Auerbach A.G., Erbes C.R., Arbisi P.A., Polusny M.A. (2011). Psychiatric diagnoses, comorbidity, and functioning in National Guard troops deployed to Iraq. J. Psychiatr. Res..

[25] Mellon S.H., Gautam A., Hammamieh R., Jett M., Wolkowitz O.M. (2018). Metabolism, Metabolomics, and Inflammation in Posttraumatic Stress Disorder. Biol. Psychiatry.

[26] Al-Turkait F.A., Ohaeri J.U. (2008). Psychopathological status, behavior problems, and family adjustment of Kuwaiti children whose fathers were involved in the first gulf war. Child. Adolesc. Psychiatry Ment. Health.

[27] Castro-Vale I., Severo M., Carvalho D. (2020). Lifetime PTSD is associated with impaired emotion recognition in veterans and their offspring. Psychiatry Res..

[28] Leen-Feldner E.W., Feldner M.T., Knapp A., Bunaciu L., Blumenthal H., Amstadter A.B. (2013). Offspring psychological and biological correlates of parental posttraumatic stress: Review of the literature and research agenda. Clin. Psychol. Rev..

[29] Steenkamp M.M., Litz B.T., Hoge C.W., Marmar C.R. (2015). Psychotherapy for Military-Related PTSD: A Review of Randomized Clinical Trials. JAMA.

[30] Dimaggio G. (2019). To expose or not to expose? The integrative therapist and posttraumatic stress disorder. J. Psychother. Integr..

[31] Steenkamp M.M., Litz B.T., Marmar C.R. (2020). First-line Psychotherapies for Military-Related PTSD. JAMA.

[32] Olff M., de Vries G.J., Guzelcan Y., Assies J., Gersons B.P. (2007). Changes in cortisol and DHEA plasma levels after psychotherapy for PTSD. Psychoneuroendocrinology.

[33] Yehuda R., Daskalakis N.P., Desarnaud F., Makotkine I., Lehrner A.L., Koch E., Flory J.D., Buxbaum J.D., Meaney M.J., Bierer L.M. (2013). Epigenetic Biomarkers as Predictors and Correlates of Symptom Improvement Following Psychotherapy in Combat Veterans with PTSD. Front. Psychiatry.

[34] Vinkers C.H., Geuze E., van Rooij S.J.H., Kennis M., Schur R.R., Nispeling D.M., Smith A.K., Nievergelt C.M., Uddin M., Rutten B.P.F. (2019). Successful treatment of post-traumatic stress disorder reverses DNA methylation marks. Mol. Psychiatry.

[35] Yehuda R., Pratchett L.C., Elmes M.W., Lehrner A., Daskalakis N.P., Koch E., Makotkine I., Flory J.D., Bierer L.M. (2014). Glucocorticoid-related predictors and correlates of post-traumatic stress disorder treatment response in combat veterans. Interface Focus.

[36] Wilker S., Pfeiffer A., Kolassa S., Elbert T., Lingenfelder B., Ovuga E., Papassotiropoulos A., de Quervain D., Kolassa I.T. (2014). The role of FKBP5 genotype in moderating long-term effectiveness of exposure-based psychotherapy for posttraumatic stress disorder. Transl. Psychiatry.

[37] Antoni F.A. (1986). Hypothalamic control of adrenocorticotropin secretion: Advances since the discovery of 41-residue corticotropin-releasing factor. Endocr. Rev..

[38] Gu Y., Piper W.T., Branigan L.A., Vazey E.M., Aston-Jones G., Lin L., LeDoux J.E., Sears R.M. (2020). A brainstem-central amygdala circuit underlies defensive responses to learned threats. Mol. Psychiatry.

[39] Bush D.E., Caparosa E.M., Gekker A., Ledoux J. (2010). Beta-adrenergic receptors in the lateral nucleus of the amygdala contribute to the acquisition but not the consolidation of auditory fear conditioning. Front. Behav. Neurosci..

[40] Schiff H.C., Johansen J.P., Hou M., Bush D.E., Smith E.K., Klein J.E., LeDoux J.E., Sears R.M. (2017). beta-Adrenergic Receptors Regulate the Acquisition and Consolidation Phases of Aversive Memory Formation Through Distinct, Temporally Regulated Signaling Pathways. Neuropsychopharmacology.

[41] Jedema H.P., Grace A.A. (2004). Corticotropin-releasing hormone directly activates noradrenergic neurons of the locus ceruleus recorded in vitro. J. Neurosci. Off. J. Soc. Neurosci..

[42] de Quervain D., Schwabe L., Roozendaal B. (2017). Stress, glucocorticoids and memory: Implications for treating fear-related disorders. Nat. Rev. Neurosci..

[43] Groeneweg F.L., Karst H., de Kloet E.R., Joels M. (2011). Rapid non-genomic effects of corticosteroids and their role in the central stress response. J. Endocrinol..

[44] Pape H.C., Pare D. (2010). Plastic synaptic networks of the amygdala for the acquisition, expression, and extinction of conditioned fear. Physiol. Rev..

[45] Lightman S.L., Wiles C.C., Atkinson H.C., Henley D.E., Russell G.M., Leendertz J.A., McKenna M.A., Spiga F., Wood S.A., Conway-Campbell B.L. (2008). The significance of glucocorticoid pulsatility. Eur J. Pharm..

[46] Juszczak G.R., Stankiewicz A.M. (2018). Glucocorticoids, genes and brain function. Prog. Neuropsychopharmacol. Biol. Psychiatry.

[47] Roozendaal B., McEwen B.S., Chattarji S. (2009). Stress, memory and the amygdala. Nat. Rev. Neurosci..

[48] Papadimitriou A., Priftis K.N. (2009). Regulation of the hypothalamic-pituitary-adrenal axis. Neuroimmunomodulation.

[49] Moisan M.P., Minni A.M., Dominguez G., Helbling J.C., Foury A., Henkous N., Dorey R., Beracochea D. (2014). Role of corticosteroid binding globulin in the fast actions of glucocorticoids on the brain. Steroids.

[50] Wyrwoll C.S., Holmes M.C., Seckl J.R. (2011). 11beta-hydroxysteroid dehydrogenases and the brain: From zero to hero, a decade of progress. Front. Neuroendocr..

[51] Quax R.A., Manenschijn L., Koper J.W., Hazes J.M., Lamberts S.W., van Rossum E.F., Feelders R.A. (2013). Glucocorticoid sensitivity in health and disease. Nat. Rev. Endocrinol..

[52] Vamvakopoulos N.V. (1995). Sexual dimorphism of stress response and immune/ inflammatory reaction: The corticotropin releasing hormone perspective. Mediat. Inflamm..

[53] Weiss E.L., Longhurst J.G., Mazure C.M. (1999). Childhood sexual abuse as a risk factor for depression in women: Psychosocial and neurobiological correlates. Am. J. Psychiatry.

[54] Dunlop B.W., Wong A. (2019). The hypothalamic-pituitary-adrenal axis in PTSD: Pathophysiology and treatment interventions. Prog. Neuropsychopharmacol. Biol. Psychiatry.

[55] Reul J.M., de Kloet E.R. (1985). Two receptor systems for corticosterone in rat brain: Microdistribution and differential occupation. Endocrinology.

[56] de Kloet E.R. (2014). From receptor balance to rational glucocorticoid therapy. Endocrinology.

[57] Nishi M., Kawata M. (2007). Dynamics of glucocorticoid receptor and mineralocorticoid receptor: Implications from live cell imaging studies. Neuroendocrinology.

[58] Ramamoorthy S., Cidlowski J.A. (2016). Corticosteroids: Mechanisms of Action in Health and Disease. Rheum. Dis. Clin. N. Am..

[59] Oakley R.H., Cidlowski J.A. (2011). Cellular processing of the glucocorticoid receptor gene and protein: New mechanisms for generating tissue-specific actions of glucocorticoids. J. Biol. Chem..

[60] Oakley R.H., Cidlowski J.A. (2013). The biology of the glucocorticoid receptor: New signaling mechanisms in health and disease. J. Allergy Clin. Immunol..

[61] Jiang S., Postovit L., Cattaneo A., Binder E.B., Aitchison K.J. (2019). Epigenetic Modifications in Stress Response Genes Associated With Childhood Trauma. Front. Psychiatry.

[62] Derijk R.H., Schaaf M.J., Turner G., Datson N.A., Vreugdenhil E., Cidlowski J., de Kloet E.R., Emery P., Sternberg E.M., Detera-Wadleigh S.D. (2001). A human glucocorticoid receptor gene variant that increases the stability of the glucocorticoid receptor beta-isoform mRNA is associated with rheumatoid arthritis. J. Rheumatol..

[63] Grad I., Picard D. (2007). The glucocorticoid responses are shaped by molecular chaperones. Mol. Cell Endocrinol..

[64] Vandevyver S., Dejager L., Van Bogaert T., Kleyman A., Liu Y., Tuckermann J., Libert C. (2012). Glucocorticoid receptor dimerization induces MKP1 to protect against TNF-induced inflammation. J. Clin. Investig..

[65] Groeneweg F.L., Karst H., de Kloet E.R., Joels M. (2012). Mineralocorticoid and glucocorticoid receptors at the neuronal membrane, regulators of nongenomic corticosteroid signalling. Mol. Cell Endocrinol..

[66] Atsak P., Roozendaal B., Campolongo P. (2012). Role of the endocannabinoid system in regulating glucocorticoid effects on memory for emotional experiences. Neuroscience.

[67] Vernocchi S., Battello N., Schmitz S., Revets D., Billing A.M., Turner J.D., Muller C.P. (2013). Membrane glucocorticoid receptor activation induces proteomic changes aligning with classical glucocorticoid effects. Mol. Cell Proteom. MCP.

[68] Binder E.B. (2009). The role of FKBP5, a co-chaperone of the glucocorticoid receptor in the pathogenesis and therapy of affective and anxiety disorders. Psychoneuroendocrinology.

[69] Vermeer H., Hendriks-Stegeman B.I., van der Burg B., van Buul-Offers S.C., Jansen M. (2003). Glucocorticoid-induced increase in lymphocytic FKBP51 messenger ribonucleic acid expression: A potential marker for glucocorticoid sensitivity, potency, and bioavailability. J. Clin. Endocrinol. Metab..

[70] Evans R.M. (1988). The steroid and thyroid hormone receptor superfamily. Science (NY).

[71] Palma-Gudiel H., Cordova-Palomera A., Leza J.C., Fananas L. (2015). Glucocorticoid receptor gene (NR3C1) methylation processes as mediators of early adversity in stress-related disorders causality: A critical review. Neurosci. Biobehav. Rev..

[72] Cao-Lei L., Leija S.C., Kumsta R., Wust S., Meyer J., Turner J.D., Muller C.P. (2011). Transcriptional control of the human glucocorticoid receptor: Identification and analysis of alternative promoter regions. Hum. Genet..

[73] Manenschijn L., van den Akker E.L., Lamberts S.W., van Rossum E.F. (2009). Clinical features associated with glucocorticoid receptor polymorphisms. An overview. Ann. NY Acad. Sci..

[74] van Rossum E.F., Roks P.H., de Jong F.H., Brinkmann A.O., Pols H.A., Koper J.W., Lamberts S.W. (2004). Characterization of a promoter polymorphism in the glucocorticoid receptor gene and its relationship to three other polymorphisms. Clin. Endocrinol. (Oxf.).

[75] Koper J.W., Stolk R.P., de Lange P., Huizenga N.A., Molijn G.J., Pols H.A., Grobbee D.E., Karl M., de Jong F.H., Brinkmann A.O. (1997). Lack of association between five polymorphisms in the human glucocorticoid receptor gene and glucocorticoid resistance. Hum. Genet..

[76] Russcher H., Smit P., van den Akker E.L., van Rossum E.F., Brinkmann A.O., de Jong F.H., Lamberts S.W., Koper J.W. (2005). Two polymorphisms in the glucocorticoid receptor gene directly affect glucocorticoid-regulated gene expression. J. Clin. Endocrinol. Metab..

[77] van Rossum E.F., Koper J.W., Huizenga N.A., Uitterlinden A.G., Janssen J.A., Brinkmann A.O., Grobbee D.E., de Jong F.H., van Duyn C.M., Pols H.A. (2002). A polymorphism in the glucocorticoid receptor gene, which decreases sensitivity to glucocorticoids in vivo, is associated with low insulin and cholesterol levels. Diabetes.

[78] Russcher H., van Rossum E.F., de Jong F.H., Brinkmann A.O., Lamberts S.W., Koper J.W. (2005). Increased expression of the glucocorticoid receptor-A translational isoform as a result of the ER22/23EK polymorphism. Mol. Endocrinol..

[79] Huizenga N.A., Koper J.W., De Lange P., Pols H.A., Stolk R.P., Burger H., Grobbee D.E., Brinkmann A.O., De Jong F.H., Lamberts S.W. (1998). A polymorphism in the glucocorticoid receptor gene may be associated with and increased sensitivity to glucocorticoids in vivo. J. Clin. Endocrinol. Metab..

[80] van Rossum E.F., Koper J.W., van den Beld A.W., Uitterlinden A.G., Arp P., Ester W., Janssen J.A., Brinkmann A.O., de Jong F.H., Grobbee D.E. (2003). Identification of the BclI polymorphism in the glucocorticoid receptor gene: Association with sensitivity to glucocorticoids in vivo and body mass index. Clin. Endocrinol. (Oxf.).

[81] Panarelli M., Holloway C.D., Fraser R., Connell J.M., Ingram M.C., Anderson N.H., Kenyon C.J. (1998). Glucocorticoid receptor polymorphism, skin vasoconstriction, and other metabolic intermediate phenotypes in normal human subjects. J. Clin. Endocrinol. Metab..

[82] Ackermann S., Heck A., Rasch B., Papassotiropoulos A., de Quervain D.J. (2013). The BclI polymorphism of the glucocorticoid receptor gene is associated with emotional memory performance in healthy individuals. Psychoneuroendocrinology.

[83] Kumsta R., Entringer S., Koper J.W., van Rossum E.F., Hellhammer D.H., Wust S. (2007). Sex specific associations between common glucocorticoid receptor gene variants and hypothalamus-pituitary-adrenal axis responses to psychosocial stress. Biol. Psychiatry.

[84] van den Akker E.L., Russcher H., van Rossum E.F., Brinkmann A.O., de Jong F.H., Hokken A., Pols H.A., Koper J.W., Lamberts S.W. (2006). Glucocorticoid receptor polymorphism affects transrepression but not transactivation. J. Clin. Endocrinol. Metab..

[85] Lian Y., Xiao J., Wang Q., Ning L., Guan S., Ge H., Li F., Liu J. (2014). The relationship between glucocorticoid receptor polymorphisms, stressful life events, social support, and post-traumatic stress disorder. BMC Psychiatry.

[86] Marceca C., Pfob M., Schelling G., Steinlein O.K., Eggert M. (2015). Single nucleotide polymorphism creating a variable upstream open reading frame regulates glucocorticoid receptor expression. Gene.

[87] Szczepankiewicz A., Leszczynska-Rodziewicz A., Pawlak J., Rajewska-Rager A., Dmitrzak-Weglarz M., Wilkosc M., Skibinska M., Hauser J. (2011). Glucocorticoid receptor polymorphism is associated with major depression and predominance of depression in the course of bipolar disorder. J. Affect. Disord..

[88] van West D., Van Den Eede F., Del-Favero J., Souery D., Norrback K.F., Van Duijn C., Sluijs S., Adolfsson R., Mendlewicz J., Deboutte D. (2006). Glucocorticoid receptor gene-based SNP analysis in patients with recurrent major depression. Neuropsychopharmacology.

[89] Nair S.C., Rimerman R.A., Toran E.J., Chen S., Prapapanich V., Butts R.N., Smith D.F. (1997). Molecular cloning of human FKBP51 and comparisons of immunophilin interactions with Hsp90 and progesterone receptor. Mol. Cell Biol..

[90] Reynolds P.D., Ruan Y., Smith D.F., Scammell J.G. (1999). Glucocorticoid resistance in the squirrel monkey is associated with overexpression of the immunophilin FKBP51. J. Clin. Endocrinol. Metab..

[91] Zannas A.S., Wiechmann T., Gassen N.C., Binder E.B. (2016). Gene-Stress-Epigenetic Regulation of FKBP5: Clinical and Translational Implications. Neuropsychopharmacology.

[92] Binder E.B., Salyakina D., Lichtner P., Wochnik G.M., Ising M., Putz B., Papiol S., Seaman S., Lucae S., Kohli M.A. (2004). Polymorphisms in FKBP5 are associated with increased recurrence of depressive episodes and rapid response to antidepressant treatment. Nat. Genet..

[93] Binder E.B., Bradley R.G., Liu W., Epstein M.P., Deveau T.C., Mercer K.B., Tang Y., Gillespie C.F., Heim C.M., Nemeroff C.B. (2008). Association of FKBP5 polymorphisms and childhood abuse with risk of posttraumatic stress disorder symptoms in adults. JAMA.

[94] Klengel T., Binder E.B. (2015). FKBP5 allele-specific epigenetic modification in gene by environment interaction. Neuropsychopharmacology.

[95] Amstadter A.B., Nugent N.R., Yang B.Z., Miller A., Siburian R., Moorjani P., Haddad S., Basu A., Fagerness J., Saxe G. (2011). Corticotrophin-releasing hormone type 1 receptor gene (CRHR1) variants predict posttraumatic stress disorder onset and course in pediatric injury patients. Dis. Mark..

[96] White S., Acierno R., Ruggiero K.J., Koenen K.C., Kilpatrick D.G., Galea S., Gelernter J., Williamson V., McMichael O., Vladimirov V.I. (2013). Association of CRHR1 variants and posttraumatic stress symptoms in hurricane exposed adults. J. Anxiety Disord..

[97] Halldorsdottir T., Binder E.B. (2017). Gene x Environment Interactions: From Molecular Mechanisms to Behavior. Annu. Rev. Psychol..

[98] Heim C., Bradley B., Mletzko T.C., Deveau T.C., Musselman D.L., Nemeroff C.B., Ressler K.J., Binder E.B. (2009). Effect of Childhood Trauma on Adult Depression and Neuroendocrine Function: Sex-Specific Moderation by CRH Receptor 1 Gene. Front. Behav. Neurosci..

[99] Webb L.M., Phillips K.E., Ho M.C., Veldic M., Blacker C.J. (2020). The Relationship between DNA Methylation and Antidepressant Medications: A Systematic Review. Int. J. Mol. Sci..

[100] Kumsta R. (2019). The role of epigenetics for understanding mental health difficulties and its implications for psychotherapy research. Psychol. Psychother..

[101] Howie H., Rijal C.M., Ressler K.J. (2019). A review of epigenetic contributions to post-traumatic stress disorder. Dialogues Clin. Neurosci..

[102] Daskalakis N.P., Rijal C.M., King C., Huckins L.M., Ressler K.J. (2018). Recent Genetics and Epigenetics Approaches to PTSD. Curr. Psychiatry Rep..

[103] Sweatt J.D., Meaney M.J.P., Nestler E.J., Akbarian S., Sweatt J.D., Meaney M.J.P., Nestler E.J., Akbarian S. (2013). Epigenetic Regulation in the Nervous System: Basic Mechanisms and Clinical Impact.

[104] Wei J.W., Huang K., Yang C., Kang C.S. (2017). Non-coding RNAs as regulators in epigenetics (Review). Oncol. Rep..

[105] Bohacek J., Mansuy I.M. (2015). Molecular insights into transgenerational non-genetic inheritance of acquired behaviours. Nat. Rev. Genet..

[106] Dias B.G., Maddox S., Klengel T., Ressler K.J. (2015). Epigenetic mechanisms underlying learning and the inheritance of learned behaviors. Trends Neurosci..

[107] Kappeler L., Meaney M.J. (2010). Epigenetics and parental effects. Bioessays.

[108] McGowan P.O., Sasaki A., D’Alessio A.C., Dymov S., Labonte B., Szyf M., Turecki G., Meaney M.J. (2009). Epigenetic regulation of the glucocorticoid receptor in human brain associates with childhood abuse. Nat. Neurosci..

[109] Linnstaedt S.D., Riker K.D., Rueckeis C.A., Kutchko K.M., Lackey L., McCarthy K.R., Tsai Y.H., Parker J.S., Kurz M.C., Hendry P.L. (2018). A Functional riboSNitch in the 3’ Untranslated Region of FKBP5 Alters MicroRNA-320a Binding Efficiency and Mediates Vulnerability to Chronic Post-Traumatic Pain. J. Neurosci. Off. J. Soc. Neurosci..

[110] Daskalakis N.P., Provost A.C., Hunter R.G., Guffanti G. (2018). Noncoding RNAs: Stress, Glucocorticoids, and Posttraumatic Stress Disorder. Biol. Psychiatry.

[111] Heim C., Nemeroff C.B. (2009). Neurobiology of posttraumatic stress disorder. CNS Spectr..

[112] Yehuda R., Hoge C.W., McFarlane A.C., Vermetten E., Lanius R.A., Nievergelt C.M., Hobfoll S.E., Koenen K.C., Neylan T.C., Hyman S.E. (2015). Post-traumatic stress disorder. Nat. Rev. Dis. Primers.

[113] Daskalakis N.P., Lehrner A., Yehuda R. (2013). Endocrine aspects of post-traumatic stress disorder and implications for diagnosis and treatment. Endocrinol. Metab. Clin. N. Am..

[114] Barker E.D., Walton E., Cecil C.A.M. (2018). Annual Research Review: DNA methylation as a mediator in the association between risk exposure and child and adolescent psychopathology. J. Child. Psychol. Psychiatryand Allied Discip..

[115] Kornfield S.L., Hantsoo L., Epperson C.N. (2018). What Does Sex Have to Do with It? The Role of Sex as a Biological Variable in the Development of Posttraumatic Stress Disorder. Curr. Psychiatry Rep..

[116] Olff M., Langeland W., Draijer N., Gersons B.P. (2007). Gender differences in posttraumatic stress disorder. Psychol. Bull..

[117] Quide Y., Andersson F., Dufour-Rainfray D., Descriaud C., Brizard B., Gissot V., Clery H., Carrey Le Bas M.P., Osterreicher S., Ogielska M. (2018). Smaller hippocampal volume following sexual assault in women is associated with post-traumatic stress disorder. Acta Psychiatr. Scand..

[118] Morris M.C., Compas B.E., Garber J. (2012). Relations among posttraumatic stress disorder, comorbid major depression, and HPA function: A systematic review and meta-analysis. Clin. Psychol. Rev..

[119] Yehuda R., Golier J.A., Halligan S.L., Meaney M., Bierer L.M. (2004). The ACTH response to dexamethasone in PTSD. Am. J. Psychiatry.

[120] Yehuda R., Golier J.A., Yang R.K., Tischler L. (2004). Enhanced sensitivity to glucocorticoids in peripheral mononuclear leukocytes in posttraumatic stress disorder. Biol. Psychiatry.

[121] McFarlane A.C., Barton C.A., Yehuda R., Wittert G. (2011). Cortisol response to acute trauma and risk of posttraumatic stress disorder. Psychoneuroendocrinology.

[122] Rohleder N., Wolf J.M., Wolf O.T. (2010). Glucocorticoid sensitivity of cognitive and inflammatory processes in depression and posttraumatic stress disorder. Neurosci. Biobehav. Rev..

[123] Meewisse M.L., Reitsma J.B., de Vries G.J., Gersons B.P., Olff M. (2007). Cortisol and post-traumatic stress disorder in adults: Systematic review and meta-analysis. Br. J. Psychiatry.

[124] Pan X., Wang Z., Wu X., Wen S.W., Liu A. (2018). Salivary cortisol in post-traumatic stress disorder: A systematic review and meta-analysis. BMC Psychiatry.

[125] Pan X., Kaminga A.C., Wen S.W., Wang Z., Wu X., Liu A. (2020). The 24-hour urinary cortisol in post-traumatic stress disorder: A meta-analysis. PLoS ONE.

[126] Wang L., Cao C., Wang W., Xu H., Zhang J., Deng H., Zhang X. (2015). Linking hair cortisol levels to phenotypic heterogeneity of posttraumatic stress symptoms in highly traumatized chinese women. Biol. Psychiatry.

[127] Steudte S., Kirschbaum C., Gao W., Alexander N., Schonfeld S., Hoyer J., Stalder T. (2013). Hair cortisol as a biomarker of traumatization in healthy individuals and posttraumatic stress disorder patients. Biol. Psychiatry.

[128] Sautter F.J., Bissette G., Wiley J., Manguno-Mire G., Schoenbachler B., Myers L., Johnson J.E., Cerbone A., Malaspina D. (2003). Corticotropin-releasing factor in posttraumatic stress disorder (PTSD) with secondary psychotic symptoms, nonpsychotic PTSD, and healthy control subjects. Biol. Psychiatry.

[129] Baker D.G., West S.A., Nicholson W.E., Ekhator N.N., Kasckow J.W., Hill K.K., Bruce A.B., Orth D.N., Geracioti T.D. (1999). Serial CSF corticotropin-releasing hormone levels and adrenocortical activity in combat veterans with posttraumatic stress disorder. Am. J. Psychiatry.

[130] Bremner J.D., Licinio J., Darnell A., Krystal J.H., Owens M.J., Southwick S.M., Nemeroff C.B., Charney D.S. (1997). Elevated CSF corticotropin-releasing factor concentrations in posttraumatic stress disorder. Am. J. Psychiatry.

[131] Raison C.L., Miller A.H. (2003). When not enough is too much: The role of insufficient glucocorticoid signaling in the pathophysiology of stress-related disorders. Am. J. Psychiatry.

[132] Mahan A.L., Ressler K.J. (2012). Fear conditioning, synaptic plasticity and the amygdala: Implications for posttraumatic stress disorder. Trends Neurosci..

[133] van Zuiden M., Geuze E., Willemen H.L., Vermetten E., Maas M., Amarouchi K., Kavelaars A., Heijnen C.J. (2012). Glucocorticoid receptor pathway components predict posttraumatic stress disorder symptom development: A prospective study. Biol. Psychiatry.

[134] Galatzer-Levy I.R., Steenkamp M.M., Brown A.D., Qian M., Inslicht S., Henn-Haase C., Otte C., Yehuda R., Neylan T.C., Marmar C.R. (2014). Cortisol response to an experimental stress paradigm prospectively predicts long-term distress and resilience trajectories in response to active police service. J. Psychiatr. Res..

[135] Steudte-Schmiedgen S., Stalder T., Schonfeld S., Wittchen H.U., Trautmann S., Alexander N., Miller R., Kirschbaum C. (2015). Hair cortisol concentrations and cortisol stress reactivity predict PTSD symptom increase after trauma exposure during military deployment. Psychoneuroendocrinology.

[136] Resnick H.S., Yehuda R., Pitman R.K., Foy D.W. (1995). Effect of previous trauma on acute plasma cortisol level following rape. Am. J. Psychiatry.

[137] Morris M.C., Hellman N., Abelson J.L., Rao U. (2016). Cortisol, heart rate, and blood pressure as early markers of PTSD risk: A systematic review and meta-analysis. Clin. Psychol. Rev..

[138] Levy-Gigi E., Szabo C., Kelemen O., Keri S. (2013). Association among clinical response, hippocampal volume, and FKBP5 gene expression in individuals with posttraumatic stress disorder receiving cognitive behavioral therapy. Biol. Psychiatry.

[139] Childress J.E., McDowell E.J., Dalai V.V., Bogale S.R., Ramamurthy C., Jawaid A., Kunik M.E., Qureshi S.U., Schulz P.E. (2013). Hippocampal volumes in patients with chronic combat-related posttraumatic stress disorder: A systematic review. J. Neuropsychiatry Clin. Neurosci.

[140] Etkin A., Wager T.D. (2007). Functional neuroimaging of anxiety: A meta-analysis of emotional processing in PTSD, social anxiety disorder, and specific phobia. Am. J. Psychiatry.

[141] Duncan L.E., Ratanatharathorn A., Aiello A.E., Almli L.M., Amstadter A.B., Ashley-Koch A.E., Baker D.G., Beckham J.C., Bierut L.J., Bisson J. (2018). Largest GWAS of PTSD (*n* = 20,070) yields genetic overlap with schizophrenia and sex differences in heritability. Mol. Psychiatry.

[142] Bachmann A.W., Sedgley T.L., Jackson R.V., Gibson J.N., Young R.M., Torpy D.J. (2005). Glucocorticoid receptor polymorphisms and post-traumatic stress disorder. Psychoneuroendocrinology.

[143] Hauer D., Weis F., Papassotiropoulos A., Schmoeckel M., Beiras-Fernandez A., Lieke J., Kaufmann I., Kirchhoff F., Vogeser M., Roozendaal B. (2011). Relationship of a common polymorphism of the glucocorticoid receptor gene to traumatic memories and posttraumatic stress disorder in patients after intensive care therapy. Crit. Care Med..

[144] van Zuiden M., Geuze E., Willemen H.L., Vermetten E., Maas M., Heijnen C.J., Kavelaars A. (2011). Pre-existing high glucocorticoid receptor number predicting development of posttraumatic stress symptoms after military deployment. Am. J. Psychiatry.

[145] Xie P., Kranzler H.R., Poling J., Stein M.B., Anton R.F., Farrer L.A., Gelernter J. (2010). Interaction of FKBP5 with childhood adversity on risk for post-traumatic stress disorder. Neuropsychopharmacology.

[146] Yehuda R., Cai G., Golier J.A., Sarapas C., Galea S., Ising M., Rein T., Schmeidler J., Muller-Myhsok B., Holsboer F. (2009). Gene expression patterns associated with posttraumatic stress disorder following exposure to the World Trade Center attacks. Biol. Psychiatry.

[147] Sarapas C., Cai G., Bierer L.M., Golier J.A., Galea S., Ising M., Rein T., Schmeidler J., Muller-Myhsok B., Uhr M. (2011). Genetic markers for PTSD risk and resilience among survivors of the World Trade Center attacks. Dis. Markers.

[148] van Zuiden M., Heijnen C.J., Maas M., Amarouchi K., Vermetten E., Geuze E., Kavelaars A. (2012). Glucocorticoid sensitivity of leukocytes predicts PTSD, depressive and fatigue symptoms after military deployment: A prospective study. Psychoneuroendocrinology.

[149] Boscarino J.A., Erlich P.M., Hoffman S.N., Zhang X. (2012). Higher FKBP5, COMT, CHRNA5, and CRHR1 allele burdens are associated with PTSD and interact with trauma exposure: Implications for neuropsychiatric research and treatment. Neuropsychiatr. Dis. Treat..

[150] Mehta D., Gonik M., Klengel T., Rex-Haffner M., Menke A., Rubel J., Mercer K.B., Putz B., Bradley B., Holsboer F. (2011). Using polymorphisms in FKBP5 to define biologically distinct subtypes of posttraumatic stress disorder: Evidence from endocrine and gene expression studies. Arch. Gen. Psychiatry.

[151] Zhang L., Hu X.Z., Yu T., Chen Z., Dohl J., Li X., Benedek D.M., Fullerton C.S., Wynn G., Barrett J.E. (2020). Genetic association of FKBP5 with PTSD in US service members deployed to Iraq and Afghanistan. J. Psychiatr. Res..

[152] Wang Q., Shelton R.C., Dwivedi Y. (2018). Interaction between early-life stress and FKBP5 gene variants in major depressive disorder and post-traumatic stress disorder: A systematic review and meta-analysis. J. Affect. Disord..

[153] Wolf E.J., Mitchell K.S., Logue M.W., Baldwin C.T., Reardon A.F., Humphries D.E., Miller M.W. (2013). Corticotropin releasing hormone receptor 2 (CRHR-2) gene is associated with decreased risk and severity of posttraumatic stress disorder in women. Depress. Anxiety.

[154] Heim C., Newport D.J., Heit S., Graham Y.P., Wilcox M., Bonsall R., Miller A.H., Nemeroff C.B. (2000). Pituitary-adrenal and autonomic responses to stress in women after sexual and physical abuse in childhood. JAMA.

[155] Zannas A.S., Provencal N., Binder E.B. (2015). Epigenetics of Posttraumatic Stress Disorder: Current Evidence, Challenges, and Future Directions. Biol. Psychiatry.

[156] Watkeys O.J., Kremerskothen K., Quide Y., Fullerton J.M., Green M.J. (2018). Glucocorticoid receptor gene (NR3C1) DNA methylation in association with trauma, psychopathology, transcript expression, or genotypic variation: A systematic review. Neurosci. Biobehav. Rev..

[157] Yehuda R., Flory J.D., Bierer L.M., Henn-Haase C., Lehrner A., Desarnaud F., Makotkine I., Daskalakis N.P., Marmar C.R., Meaney M.J. (2015). Lower methylation of glucocorticoid receptor gene promoter 1F in peripheral blood of veterans with posttraumatic stress disorder. Biol. Psychiatry.

[158] Vukojevic V., Kolassa I.T., Fastenrath M., Gschwind L., Spalek K., Milnik A., Heck A., Vogler C., Wilker S., Demougin P. (2014). Epigenetic modification of the glucocorticoid receptor gene is linked to traumatic memory and post-traumatic stress disorder risk in genocide survivors. J. Neurosci. Off. J. Soc. Neurosci..

[159] Perroud N., Rutembesa E., Paoloni-Giacobino A., Mutabaruka J., Mutesa L., Stenz L., Malafosse A., Karege F. (2014). The Tutsi genocide and transgenerational transmission of maternal stress: Epigenetics and biology of the HPA axis. World J. Biol. Psychiatry.

[160] Labonte B., Azoulay N., Yerko V., Turecki G., Brunet A. (2014). Epigenetic modulation of glucocorticoid receptors in posttraumatic stress disorder. Transl. Psychiatry.

[161] Schechter D.S., Moser D.A., Paoloni-Giacobino A., Stenz L., Gex-Fabry M., Aue T., Adouan W., Cordero M.I., Suardi F., Manini A. (2015). Methylation of NR3C1 is related to maternal PTSD, parenting stress and maternal medial prefrontal cortical activity in response to child separation among mothers with histories of violence exposure. Front. Psychol..

[162] Yehuda R., Daskalakis N.P., Bierer L.M., Bader H.N., Klengel T., Holsboer F., Binder E.B. (2016). Holocaust Exposure Induced Intergenerational Effects on FKBP5 Methylation. Biol. Psychiatry.

[163] Kang J.I., Kim T.Y., Choi J.H., So H.S., Kim S.J. (2019). Allele-specific DNA methylation level of FKBP5 is associated with post-traumatic stress disorder. Psychoneuroendocrinology.

[164] Lebow M.A., Schroeder M., Tsoory M., Holzman-Karniel D., Mehta D., Ben-Dor S., Gil S., Bradley B., Smith A.K., Jovanovic T. (2019). Glucocorticoid-induced leucine zipper “quantifies” stressors and increases male susceptibility to PTSD. Transl. Psychiatry.

[165] Rutten B.P.F., Vermetten E., Vinkers C.H., Ursini G., Daskalakis N.P., Pishva E., de Nijs L., Houtepen L.C., Eijssen L., Jaffe A.E. (2018). Longitudinal analyses of the DNA methylome in deployed military servicemen identify susceptibility loci for post-traumatic stress disorder. Mol. Psychiatry.

[166] Mehta D., Voisey J., Bruenig D., Harvey W., Morris C.P., Lawford B., Young R.M. (2018). Transcriptome analysis reveals novel genes and immune networks dysregulated in veterans with PTSD. Brainbehav. Immun..

[167] Kumsta R., Marzi S.J., Viana J., Dempster E.L., Crawford B., Rutter M., Mill J., Sonuga-Barke E.J. (2016). Severe psychosocial deprivation in early childhood is associated with increased DNA methylation across a region spanning the transcription start site of CYP2E1. Transl. Psychiatry.

[168] Tylee D.S., Kawaguchi D.M., Glatt S.J. (2013). On the outside, looking in: A review and evaluation of the comparability of blood and brain “-omes”. Am. J. Med. Genet. B Neuropsychiatr. Genet..

[169] Szyf M., Bick J. (2013). DNA methylation: A mechanism for embedding early life experiences in the genome. Child. Dev..

[170] Schelling G., Briegel J., Roozendaal B., Stoll C., Rothenhausler H.B., Kapfhammer H.P. (2001). The effect of stress doses of hydrocortisone during septic shock on posttraumatic stress disorder in survivors. Biol. Psychiatry.

[171] Schelling G., Kilger E., Roozendaal B., de Quervain D.J., Briegel J., Dagge A., Rothenhausler H.B., Krauseneck T., Nollert G., Kapfhammer H.P. (2004). Stress doses of hydrocortisone, traumatic memories, and symptoms of posttraumatic stress disorder in patients after cardiac surgery: A randomized study. Biol. Psychiatry.

[172] Schelling G., Roozendaal B., Krauseneck T., Schmoelz M., De Quervain D., Briegel J. (2006). Efficacy of hydrocortisone in preventing posttraumatic stress disorder following critical illness and major surgery. Ann. NY Acad. Sci..

[173] Zohar J., Yahalom H., Kozlovsky N., Cwikel-Hamzany S., Matar M.A., Kaplan Z., Yehuda R., Cohen H. (2011). High dose hydrocortisone immediately after trauma may alter the trajectory of PTSD: Interplay between clinical and animal studies. Eur. Neuropsychopharmacol..

[174] Delahanty D.L., Gabert-Quillen C., Ostrowski S.A., Nugent N.R., Fischer B., Morris A., Pitman R.K., Bon J., Fallon W. (2013). The efficacy of initial hydrocortisone administration at preventing posttraumatic distress in adult trauma patients: A randomized trial. CNS Spectr..

[175] Aerni A., Traber R., Hock C., Roozendaal B., Schelling G., Papassotiropoulos A., Nitsch R.M., Schnyder U., de Quervain D.J. (2004). Low-dose cortisol for symptoms of posttraumatic stress disorder. Am. J. Psychiatry.

[176] Dunlop B.W., Binder E.B., Iosifescu D., Mathew S.J., Neylan T.C., Pape J.C., Carrillo-Roa T., Green C., Kinkead B., Grigoriadis D. (2017). Corticotropin-Releasing Factor Receptor 1 Antagonism Is Ineffective for Women With Posttraumatic Stress Disorder. Biol. Psychiatry.

[177] Ludascher P., Schmahl C., Feldmann R.E., Kleindienst N., Schneider M., Bohus M. (2015). No evidence for differential dose effects of hydrocortisone on intrusive memories in female patients with complex post-traumatic stress disorder—A randomized, double-blind, placebo-controlled, crossover study. J. Psychopharmacol..

[178] Golier J.A., Yehuda R., Baker D. (2017). A randomized clinical trial of a glucocorticoid receptor antagonist in PTSD. Psychoneuroendocrinology.

[179] Yehuda R., Bierer L.M., Pratchett L.C., Lehrner A., Koch E.C., Van Manen J.A., Flory J.D., Makotkine I., Hildebrandt T. (2015). Cortisol augmentation of a psychological treatment for warfighters with posttraumatic stress disorder: Randomized trial showing improved treatment retention and outcome. Psychoneuroendocrinology.

[180] Galatzer-Levy I.R., Andero R., Sawamura T., Jovanovic T., Papini S., Ressler K.J., Norrholm S.D. (2017). A cross species study of heterogeneity in fear extinction learning in relation to FKBP5 variation and expression: Implications for the acute treatment of posttraumatic stress disorder. Neuropharmacology.

[181] Pape J.C., Carrillo-Roa T., Rothbaum B.O., Nemeroff C.B., Czamara D., Zannas A.S., Iosifescu D., Mathew S.J., Neylan T.C., Mayberg H.S. (2018). DNA methylation levels are associated with CRF1 receptor antagonist treatment outcome in women with post-traumatic stress disorder. Clin. Epigenet..

[182] Ostacher M.J., Cifu A.S. (2019). Management of Posttraumatic Stress Disorder. JAMA.

[183] Watkins L.E., Sprang K.R., Rothbaum B.O. (2018). Treating PTSD: A Review of Evidence-Based Psychotherapy Interventions. Front. Behav. Neurosci..

[184] Brom D., Kleber R.J., Defares P.B. (1989). Brief psychotherapy for posttraumatic stress disorders. J. Consult. Clin. Psychol..

[185] Gerber A.J., Kocsis J.H., Milrod B.L., Roose S.P., Barber J.P., Thase M.E., Perkins P., Leon A.C. (2011). A quality-based review of randomized controlled trials of psychodynamic psychotherapy. Am. J. Psychiatry.

[186] Thoma N.C., McKay D., Gerber A.J., Milrod B.L., Edwards A.R., Kocsis J.H. (2012). A quality-based review of randomized controlled trials of cognitive-behavioral therapy for depression: An assessment and metaregression. Am. J. Psychiatry.

[187] Cordioli A.V., Cordioli A.V. (2008). As principais psicoterapias: Fundamentos teóricos, técnicas, indicações e contra-indicações. Psicoterapias: Abordagens Atuais.

[188] Lambert M.J. (2013). Outcome in psychotherapy: The past and important advances. Psychotherapy.

[189] Roth A., Fonagy P. (2005). What Works for Whom? A Critical Review of Psychotherapy Research.

[190] Daskalakis N.P., Bagot R.C., Parker K.J., Vinkers C.H., de Kloet E.R. (2013). The three-hit concept of vulnerability and resilience: Toward understanding adaptation to early-life adversity outcome. Psychoneuroendocrinology.

[191] Nederhof E., Schmidt M.V. (2012). Mismatch or cumulative stress: Toward an integrated hypothesis of programming effects. Physiol. Behav..

[192] Yehuda R., LeDoux J. (2007). Response variation following trauma: A translational neuroscience approach to understanding PTSD. Neuron.

[193] Meir Drexler S., Wolf O.T. (2017). The role of glucocorticoids in emotional memory reconsolidation. Neurobiol. Learn. Mem..

[194] Rapcencu A.E., Gorter R., Kennis M., van Rooij S.J.H., Geuze E. (2017). Pre-treatment cortisol awakening response predicts symptom reduction in posttraumatic stress disorder after treatment. Psychoneuroendocrinology.

[195] Rauch S.A., King A.P., Abelson J., Tuerk P.W., Smith E., Rothbaum B.O., Clifton E., Defever A., Liberzon I. (2015). Biological and symptom changes in posttraumatic stress disorder treatment: A randomized clinical trial. Depress. Anxiety.

[196] Dajani R., Hadfield K., van Uum S., Greff M., Panter-Brick C. (2018). Hair cortisol concentrations in war-affected adolescents: A prospective intervention trial. Psychoneuroendocrinology.

[197] Steudte-Schmiedgen S., Kirschbaum C., Alexander N., Stalder T. (2016). An integrative model linking traumatization, cortisol dysregulation and posttraumatic stress disorder: Insight from recent hair cortisol findings. Neurosci. Biobehav. Rev..

[198] Roozendaal B., Hahn E.L., Nathan S.V., de Quervain D.J., McGaugh J.L. (2004). Glucocorticoid effects on memory retrieval require concurrent noradrenergic activity in the hippocampus and basolateral amygdala. J. Neurosci. Off. J. Soc. Neurosci..

[199] de Quervain D.J., Aerni A., Schelling G., Roozendaal B. (2009). Glucocorticoids and the regulation of memory in health and disease. Front. Neuroendocr..

[200] Quide Y., Witteveen A.B., El-Hage W., Veltman D.J., Olff M. (2012). Differences between effects of psychological versus pharmacological treatments on functional and morphological brain alterations in anxiety disorders and major depressive disorder: A systematic review. Neurosci. Biobehav. Rev..

[201] Butler O., Willmund G., Gleich T., Gallinat J., Kuhn S., Zimmermann P. (2018). Hippocampal gray matter increases following multimodal psychological treatment for combat-related post-traumatic stress disorder. Brain Behav..

[202] Sapolsky R.M., Romero L.M., Munck A.U. (2000). How do glucocorticoids influence stress responses? Integrating permissive, suppressive, stimulatory, and preparative actions. Endocr. Rev..

[203] Eriksson P.S., Perfilieva E., Bjork-Eriksson T., Alborn A.M., Nordborg C., Peterson D.A., Gage F.H. (1998). Neurogenesis in the adult human hippocampus. Nat. Med..

[204] Margraf J., Zlomuzica A. (2015). Changing the future, not the past: A translational paradigm shift in treating anxiety. EMBO Rep..

[205] Kandel E.R. (1998). A new intellectual framework for psychiatry. Am. J. Psychiatry.

[206] Hauer D., Kolassa I.T., Laubender R.P., Mansmann U., Hagl C., Roozendaal B., de Quervain D.J., Schelling G. (2013). A genotype-specific, randomized controlled behavioral intervention to improve the neuroemotional outcome of cardiac surgery: Study protocol for a randomized controlled trial. Trials.

[207] Vinkers C., Geuze E., van Rooij S., Kennis M., Schur R., Nispeling D., Smith A., Nievergelt C., Uddin M., Rutten B. (2019). O41. Longitudinal Changes in Genome-Wide DNA Methylation Levels Related to Treatment Outcomes and Recovery From Post-Traumatic Stress Disorder. Biol. Psychiatry.

[208] Jakobsson J., Cordero M.I., Bisaz R., Groner A.C., Busskamp V., Bensadoun J.C., Cammas F., Losson R., Mansuy I.M., Sandi C. (2008). KAP1-mediated epigenetic repression in the forebrain modulates behavioral vulnerability to stress. Neuron.

[209] Feodorova Y.N., Sarafian V.S. (2012). Psychological stress—cellular and molecular mechanisms. Folia Med. (Plovdiv).

[210] Zerach G., Kanat-Maymon Y., Aloni R., Solomon Z. (2016). The role of fathers’ psychopathology in the intergenerational transmission of captivity trauma: A twenty three-year longitudinal study. J. Affect. Disord..

[211] Lang A.J., Gartstein M.A. (2018). Intergenerational transmission of traumatization: Theoretical framework and implications for prevention. J. Trauma Dissociation Off. J. Int. Soc. Study Dissociation.

[212] Gentile S. (2015). Early pregnancy exposure to selective serotonin reuptake inhibitors, risks of major structural malformations, and hypothesized teratogenic mechanisms. Exp. Opin. Drug Metab. Toxicol..

[213] Tomaz T., Castro-Vale I. (2020). Trauma-Informed Care in Primary Health Settings-Which Is Even More Needed in Times of COVID-19. Healthcare (Basel).

